# Association Between Genetic Risk, Adherence to Healthy Lifestyle Behavior, and Thyroid Cancer Risk

**DOI:** 10.1001/jamanetworkopen.2022.46311

**Published:** 2022-12-12

**Authors:** Xiuming Feng, Fei Wang, Wenjun Yang, Yuan Zheng, Chaoqun Liu, Lulu Huang, Longman Li, Hong Cheng, Haiqing Cai, Xiangzhi Li, Xing Chen, Xiaobo Yang

**Affiliations:** 1 Department of Occupational Health and Environmental Health, School of Public Health, Guangxi Medical University, Nanning, Guangxi, China; 2 Center for Genomic and Personalized Medicine, Guangxi Key Laboratory for Genomic and Personalized Medicine, Guangxi Collaborative Innovation Center for Genomic and Personalized Medicine, Guangxi Medical University, Nanning, Guangxi, China; 3 Guangxi Key Laboratory on Precise Prevention and Treatment for Thyroid Tumor, Guangxi University of Science and Technology, Liuzhou, Guangxi, China; 4 Department of Nutrition and Food Hygiene, School of Public Health, Guangxi Medical University, Nanning, Guangxi, China; 5 Department of Radiotherapy, First Affiliated Hospital of Guangxi Medical University, Nanning, Guangxi, China; 6 Department of Urology, Institute of Urology and Nephrology, First Affiliated Hospital of Guangxi Medical University, Nanning, Guangxi, China; 7 Department of Public Health, School of Medicine, Guangxi University of Science and Technology, Liuzhou, Guangxi, China; 8 Department of Sanitary Chemistry, School of Public Health, Guangxi Medical University, Nanning, Guangxi, China; 9 Guangxi Key Laboratory of Environment and Health Research, Department of Occupational Health and Environmental Health, School of Public Health, Guangxi Medical University, Nanning, Guangxi, China

## Abstract

**Importance:**

Genetic and lifestyle factors are related to thyroid cancer (TC). Whether a healthy lifestyle is associated with TC and could attenuate the influence of genetic variants in TC remains equivocal.

**Objectives:**

To examine the associations between genetics and healthy lifestyle with incident TC and whether adherence to a healthy lifestyle modifies the association between genetic variants and TC.

**Design, Setting, and Participants:**

A prospective cohort study using UK Biobank data recruited 502 505 participants aged 40 to 69 years between March 13, 2006, and October 1, 2010. A total of 307 803 participants of European descent were recruited at baseline, and 264 956 participants were available for the present study. Data analysis was conducted from November 1, 2021, to April 22, 2022.

**Exposures:**

Lifestyle behaviors were determined by diet index, physical activity, weight, smoking, and alcohol consumption. Lifestyle was categorized as unfavorable (scores 0-1), intermediate (score 2), and favorable (scores 3-5). The polygenic risk score (PRS) was derived from a meta–genome-wide association study using 3 cohorts and categorized as low, intermediate, and high.

**Main Outcomes and Measures:**

Thyroid cancer was defined using the *International Classification of Diseases, Ninth Revision* (code 193), *International Statistical Classification of Diseases and Related Health Problems, Tenth Revision* (code C73), and self-report (code 1065).

**Results:**

Of 264 956 participants, 137 665 were women (52%). The median age was 57 (IQR, 49-62) years. During a median follow-up of 11.1 (IQR, 10.33-11.75) years (2 885 046 person-years), 423 incident TCs were ascertained (14.66 per 100 000 person-years). Higher PRSs were associated with TC (hazard ratio [HR], 2.25; 95% CI, 1.91-2.64; *P* = 8.65 × 10^−23^). An unfavorable lifestyle was also associated with a higher risk of TC (HR, 1.93; 95% CI, 1.50-2.49; *P* < .001). When stratified by PRS, unfavorable lifestyle was associated with TC in the higher PRS group (favorable vs unfavorable HR, 0.52; 95% CI, 0.37-0.73; *P* < .001). Furthermore, participants with both a high PRS and unfavorable lifestyle had the highest risk of TC (HR, 4.89; 95% CI, 3.03-7.91; *P* < .001).

**Conclusions and Relevance:**

In this prospective cohort study, genetic and lifestyle factors were independently associated with incident TC, which suggests that a healthier lifestyle may attenuate the deleterious influence of genetics on the risk of TC in individuals of European descent.

## Key Points

**Question:**

Is a healthy lifestyle associated with thyroid cancer risk, and could it attenuate the influence of genetic variants on thyroid cancer?

**Findings:**

In this cohort study that included 423 patients with incident thyroid cancer and 264 533 individuals without thyroid cancer, adherence to a healthier lifestyle attenuated the negative association of genetic factors with the risk of thyroid cancer in those of European descent. Participants with both a high polygenic risk score and an unfavorable lifestyle had the highest risk of thyroid cancer.

**Meaning:**

The findings of this study highlight the potential of lifestyle interventions to reduce the risk of thyroid cancer, even in those at high genetic risk.

## Teaser

This cohort study examines whether a healthy lifestyle affects the association between genetic variants and risk of thyroid cancer.

## Introduction

The increasing incidence and financial burden of thyroid cancer (TC) have drawn widespread attention. Its incidence has increased by approximately 10% annually over the past 30 years.^1^ According to Global Cancer Statistics, TC ranks ninth among 36 cancers globally.^2^ Based on the US health care system, the total cumulative costs incurred by a TC diagnosis increased to more than $2.38 billion in 2019.^3^

Cancer development is related to genetic and environmental factors. The heritability of TC may be approximately 50%, which is the highest among the 15 most common cancers.^4^ Recent genome-wide association studies (GWASs) have identified several single nucleotide variants (SNVs) that are substantially noted in TC.^5^ A multicenter study^6^ performed in patients of European descent also reported that a higher polygenic risk score (PRS) was associated with an increased risk of TC.

Lifestyles are important modifiable environmental factors in the development of various cancers. However, convincing and consistent evidence regarding the association between lifestyle and TC is lacking. Recently, studies^7,8^ combined 5 to 7 lifestyle behaviors (diet, physical activity, alcohol use, smoking, and weight) to synthetically estimate their association with breast cancer, ovarian cancer, and colorectal cancer and have reported concordant results that healthy lifestyles could alleviate the risk of cancer. However, few studies have investigated multiple lifestyle factors regarding TC. Moreover, the findings on physical activity,^9^ alcohol use,^10,11^ smoking,^10,11^ and diet^12,13,14^ and TC risk were inconsistent except for obesity.^15,16,17^ Furthermore, previous studies^18,19,20,21^ combined genetics and lifestyle factors to explore their association with cancers and discovered that a healthy lifestyle could alleviate the risk of cancer (eg, breast cancer and gastric cancer) due to high genetic predispositions.

To our knowledge, no comprehensive study has examined the interaction and joint association between genetics and lifestyle and the risk of TC. Therefore, our research aimed to explore whether PRS and a healthy lifestyle are associated with TC in participants of European descent in the UK Biobank. We further investigated whether lifestyle influenced the risk of TC in those with high genetic predispositions.

## Methods

### Study Population

The UK Biobank, a large-scale prospective cohort containing in-depth genetic and detailed health-related data and lifestyle information, provides a platform for integrative analyses of genetic variation, modifiable risk factors, and a wide range of diseases, including cancers. Detailed information regarding the UK Biobank is described elsewhere.^22^ Briefly, 502 505 participants aged 40 to 69 years were enrolled at baseline covering 22 assessment centers between March 13, 2006, and October 1, 2010. Following the standardized process of interviews and questionnaires, anthropometric indices (height, body mass index, waist circumference, blood pressure, heart rate, and other measures), lifestyle, and environmental factors (diet, exercise, smoking, alcohol use, and other factors) were collected in the last main-stage assessment centers. The quality of the interviews and questionnaires was validated by trained staff and then uploaded to the resource acquisition system. This cohort study was approved by the relevant ethical committees for the UK Biobank. All participants provided written informed consent. This study followed the Strengthening the Reporting of Observational Studies in Epidemiology (STROBE) reporting guideline.

eFigure 1 in the Supplement presents an overview of the study design. Of the initial cohort of 502 505 participants, 475 653 individuals were of European descent (including British, Irish, White, White and Asian, and White and Black African). We deleted 40 951 individuals who were diagnosed with cancer prior to enrollment and individuals (n = 126 899) with covariates missing (n = 6709), lifestyle variables missing (n = 54 009), genetic information missing (n = 35 226), and nonconformity between genetic sex, race and ethnicity, and self-reported sex and ethnicity (n = 30 955). With these exclusions, there were 307 803 participants available for the study. The complete follow-up period was up to February 29, 2020, for England and Wales and October 31, 2015, for Scotland. During the follow-up period, 42 847 participants with incident cancer other than TC were excluded. For the subsequent analysis, we included 423 participants with incident TC and 264 533 individuals without TC.

### Diagnosis of TC

Data on the diagnosis of TC were linked to the *International Classification of Diseases, Ninth Revision* (*ICD-9*) and *International Statistical Classification of Diseases and Related Health Problems, Tenth Revision* (*ICD-10*), self-reported cancer, and surgery. The TC code was derived from 3 databases (self-report code 1065, *ICD-9* code 193, and *ICD-10* code C73).

### GWAS and PRS for TC

The GWAS was conducted in 2 steps: the first was performed on the participants of the UK Biobank, and the second was a meta-GWAS using 3 cohorts (UK Biobank, FinnGen Study, and Italian residents). Detailed genotyping information for the UK Biobank is available.^23^ Samples were genotyped using the UK Biobank Axiom Array and UK BiLEVE Axiom Array.^24^ The released genotype data were imputed with reference to the haplotype reference consortium panel. GWAS Manhattan and quantile-quantile plots were produced and checked for each variant. The genomic inflation factor was calculated using linkage disequilibrium score regression analysis.^25^ Detailed information on the quality control procedures of the GWAS and meta-GWAS is provided in eMethods 1 in the Supplement. Based on the results of the meta-analysis, we established PRSs using SNVs at *P* < 5 × 10^−5^. We calculated PRSs by summing the product of the risk variant and the corresponding estimate across the meta-GWAS results.^26^ Polygenic risk scores were divided into low (bottom tertiles), intermediate (middle tertiles), and high (top tertiles) genetic risk, as described previously.^26^ All SNVs were abstracted from the autosome, with *r*^2^ less than 0.99 and minor allele frequency greater than or equal to 0.01.

### Lifestyle Behaviors

Five lifestyle behaviors were available to construct the total lifestyle: diet index (comprising fruits and vegetables, fish, red meat and processed meats, whole grains, refined grains, and sugar-sweetened beverages), total moderate to vigorous physical activity, weight (consisting of body mass index and waist circumference), smoking, and alcohol consumption. Each variable was assigned a score of 0 or 1, with 1 representing a favorable lifestyle behavior (listed in eTable 1 in the Supplement). To capture a more detailed spectrum of each lifestyle behavior, we performed sex-specific Cox proportional hazards models to obtain estimates (log_e_ hazard ratio [HR]) for each lifestyle component. The weighted lifestyle was calculated as:
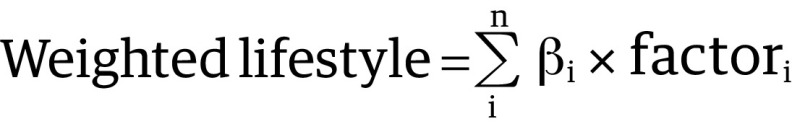
where *β_i_* is the sex-specific estimate for each lifestyle component, *factor_i_* is each lifestyle behavior, and there are 5 lifestyle behaviors. Thus, 2 lifestyles (unweighted and weighted) were created, and the weighted lifestyle resembled the unweighted lifestyle distribution.

### Covariate Definition

Educational level qualifications were categorized as college or university degree; secondary education, including A levels/AS levels or equivalent, O levels/general certificate of secondary education or equivalent, and certificates of secondary education or equivalent; and some professional qualifications, including national vocational qualification, higher national diplomas, higher national certificates, or equivalent and other professional qualifications.^21^ Socioeconomic status was derived from the Townsend deprivation index quintiles (1, 2-4, and 5). The average total household income before tax was divided into 5 groups: less than £18 000, £18 000 to £30 999, £31 000 to £51 999, £52 000 to £100 000, and greater than £100 000 (exchange rate in November 2022 of $1.00 = £0.88). The dichotomous variables were ever having taken the oral contraceptive pill, ever having used hormone-replacement therapy, and menopausal status. Menopausal status was classified as premenopausal and postmenopausal. Women with missing data on menopausal status were categorized as postmenopausal with a surgical history of bilateral oophorectomy (or hysterectomy) or age older than 55 years.^27^

### Statistical Analysis

Categorical variables are reported as numbers and percentages, normally distributed continuous variables as mean (SD), and skewed distributed variables as median (IQR). The comparison between the 2 groups was estimated using the Pearson χ^2^ test or Mann-Whitney test.

Cox proportional hazards models were used to assess the association between the PRS (continuous and tertiles), lifestyle, and TC, and the interaction between lifestyle and PRS on the risk of TC. *P* values for trend were estimated, using PRS and lifestyle factors as continuous variables. Stratification analysis of the PRS and lifestyle was performed (unfavorable lifestyle and lowest PRS as the reference). The multiplicative interaction was calculated by modeling a multiplicative term between the PRS and lifestyle in the models, and the additive interaction between the PRS and lifestyle was multiplied by using relative excess risk (RERI).^28^ The main analyses were adjusted for age, sex, the first 5 genetic principal components, educational qualifications, socioeconomic status, and the average total household income before tax. The women category was further adjusted for age at menarche, menopausal status, number of live births, use of oral contraceptive pills, and use of hormone replacement therapy.

In the sensitivity analysis, we performed 5 approaches: (1) computed 4 PRSs (a brief procedure for constructing PRSs is described in eMethods 2 and eTable 2 in the Supplement) and evaluated the predictive performance of the PRS using receiver operating characteristic curves; (2) established weighted and unweighted PRS and lifestyle; (3) conducted a 1:5 matching nested case-control study, with the pairing factors sex, age (±5 years), race and ethnicity, assessment centers, time of enrollment, and relevant analysis using conditional logistic regression; (4) applied a sex stratification analysis; and (5) conducted competing risk analysis (set the cancer cases or deaths or loss during follow-up as the competing event).

GWASs were conducted using Plink2, R software, version 4.4 (R Foundation for Statistical Computing) was used to perform conditional logistic regression analyses and Cox proportional hazards models (the package survival), the meta-GWAS (the package rmeta), additive interaction (interaction R). Statistical power was calculated using an online power calculator.^29^ We considered 2-sided *P* values <.05 as statistically significant. Data analysis was conducted from November 1, 2021, to April 22, 2022.

## Results

### The Population of TC

Of 264 956 participants included in our study, there were 137 665 women and 127 291 men. The median age was 57 (IQR, 49-62) years. During a median follow-up of 11.1 (IQR, 10.33-11.75) years (2 885 046 person-years), there were 423 cases of incident TC with an incidence rate of 14.66 per 100 000 person-years in the total population. The incidence rate was almost 3-fold higher in women than in men (20.2 vs 8.7 per 100 000 person-years). The proportion of unfavorable weighted lifestyle was more evident in the group with incident TC (39.95% vs 33.59%; *P* < .001) compared with participants without TC (Table 1; eTable 3 in the Supplement). Compared with participants without TC, those with TC had a more unfavorable lifestyle component, including moderate to vigorous physical activity (55.56% vs 46.17%), weight (63.36% vs 55.27%), and smoking (10.87% vs 7.83%). Differences in 5 lifestyle factors and total lifestyle were significant between men and women (eTable 4 in the Supplement). Men had more a unfavorable diet index (54.15% vs 39.22%), weight (57.78% vs 52.97%), smoke intake (9.76% vs 6.05%), alcohol consumption (31.82% vs 29.89%), and weighted lifestyle (46.39% vs 21.78%) compared with women. Women had more unfavorable moderate to vigorous physical activity (47.68% vs 44.57%).

**Table 1.** Baseline Characteristics of Participants of TC in the UK Biobank

Variable	No. (%)			*P* value
	Overall (N = 264 956)	Nonincident TC (n = 264 533)	Incident TC (n = 423)	
Age, median (IQR), y	57 (49-62)	57 (49-62)	59 (51-63)	<.001
Sex				
Women	137 665 (51.96)	137 363 (51.93)	302 (71.39)	<.001
Men	127 291 (48.04)	127 170 (48.07)	121 (28.61)	
Townsend deprivation index				
1 (Least deprived)	58 903 (22.23)	58 817 (22.23)	86 (20.33)	.15
2-4	164 657 (62.15)	164 400 (62.15)	257 (60.76)	
5 (Most deprived)	41 396 (15.62)	41 316 (15.62)	80 (18.91)	
Educational qualifications				
College or university degree	91 393 (34.49)	91 256 (34.50)	137 (32.39)	.26
Secondary education	143 476 (54.15)	143 231 (54.14)	245 (57.92)	
Some professional qualifications	30 087 (11.36)	30 046 (11.36)	41 (9.69)	
Average total household income before tax, £ ^a^				
<18 000	43 803 (16.53)	43 724 (16.53)	79 (18.68)	<.001
18 000-30 999	57 684 (21.77)	57 585 (21.77)	99 (23.40)	
31 000-51 999	95 208 (35.93)	95 028 (35.92)	180 (42.55)	
52 000-100 000	54 069 (20.41)	54 015 (20.42)	54 (12.77)	
>100 000	14 192 (5.36)	14 181 (5.36)	11 (2.60)	
Diet index				
Unfavorable	122 919 (46.39)	122 734 (46.40)	185 (43.74)	.30
Favorable	142 037 (53.61)	141 799 (53.60)	238 (56.26)	
Total moderate to vigorous physical activity				
Unfavorable	122 370 (46.19)	122 135 (46.17)	235 (55.56)	<.001
Favorable	142 586 (53.81)	142 398 (53.83)	188 (44.44)	
Healthy weight				
Unfavorable	146 465 (55.28)	146 197 (55.27)	268 (63.36)	.001
Favorable	118 491 (44.72)	118 336 (44.73)	155 (36.64)	
Smoke intake				
Unfavorable	20 757 (7.83)	20 711 (7.83)	46 (10.87)	.03
Favorable	244 199 (92.17)	243 822 (92.17)	377 (89.13)	
Alcohol consumption				
Unfavorable	81 653 (30.82)	81 560 (30.83)	93 (21.75)	<.001
Favorable	183 303 (69.18)	182 973 (69.17)	330 (78.25)	
Lifestyle				
Unfavorable	74 852 (28.25)	74 717 (28.25)	135 (31.91)	.14
Intermediate	87 687 (33.10)	87 545 (33.09)	142 (33.57)	
Favorable	102 417 (38.65)	102 271 (38.66)	146 (34.52)	
Weighted lifestyle				
Favorable	94 006 (35.48)	93 906 (35.50)	95 (22.46)	<.001
Intermediate	81 915 (30.92)	81 759 (30.91)	159 (37.59)	
Unfavorable	89 035 (33.60)	88 868 (33.59)	169 (39.95)	

Abbreviation: TC, thyroid cancer.

^a^ Exchange rate November 2022 of $1.00 = £0.88.

### GWAS Analysis, PRS Selection, and Comparison

We conducted the GWAS and meta-analysis based on 3 cohorts. The detailed results of the GWAS are listed in the eAppendix, eTable 5, eTable 6, and eFigure 2 in the Supplement. According to the results of the meta-GWAS, 15 SNVs were used to establish the first PRS (PRS1) (related SNVs are presented in eTable 7 in the Supplement). We found that the PRS as a continuous variable was associated with increased risk of TC (HR, 2.25; 95% CI, 1.91-2.64; *P* = 8.65 × 10^−23^). Table 2 presents a gradual increased association noted between the PRS and TC risk. Individuals with a high PRS had a 2.82-fold risk associated with increased TC risk (95% CI, 2.16-3.68; *P* = 2.34 × 10^−14^), whereas those with intermediate PRS had 1.71-fold risk associated with increased TC risk (95% CI, 1.28-2.28, *P* = 2.56 × 10^−4^) compared with the lowest PRS. Consistent results were observed with the other 3 PRS categories (eTable 8 in the Supplement). Furthermore, we applied an area under the receiver operating characteristic curve to evaluate the power of the PRSs (eFigure 3 in the Supplement). The receiver operating characteristic curves for PRS1 had better performance and a small number of SNVs (PRS1: 0.63; 95% CI, 0.61-0.66; PRS2: 0.62; 95% CI, 0.59-0.64; PRS3: 0.64; 95% CI, 0.61-0.66; and PRS4: 0.62; 95% CI, 0.59-0.64). Combining the performance results and estimate, PRS1 was used in the consequent assessment. In addition, we achieved more than 80% power using PRS1, with an odds ratio greater than 1.30.

**Table 2.** Association Between PRS and Risk of TC

Characteristic	TC/non-TC	Model 1^a^				Model 2^a^			
		HR (95% CI)	*P* value	HR (95% CI)^b^	*P* value^b^	HR (95% CI)	*P* value	HR (95% CI)^b^	*P* value^b^
Weighted PRS1 (15 SNVs)^c^	NA	NA	NA	2.48 (1.74-3.53)	4.77 × 10^−7^	NA	NA	2.25 (1.91-2.64)	8.65 × 10^−23^
Low	75/88 248	1 [Reference]	NA	NA	NA	1 [Reference]	NA	NA	NA
Intermediate	132/88 182	1.76 (1.33-2.34)	9.10 × 10^−5^	NA	NA	1.71 (1.28-2.28)	2.56 × 10^−4^	NA	NA
High	216/88 103	2.89 (2.22-3.75)	2.64 × 10^−15^	NA	NA	2.82 (2.16-3.68)	2.34 × 10^−14^	NA	NA
Unweighted PRS1 (15 SNVs)^c^	NA	NA	NA	1.04 (1.00-1.09)	.04	NA	NA	1.03 (0.99-1.07)	.15
Low	116/79 711	1 [Reference]		NA	NA	1 [Reference]	NA	NA	NA
Intermediate	137/87 980	1.07 (0.84-1.37)	.59	NA	NA	1.11 (0.87-1.43)	.40	NA	NA
High	170/96 842	1.21 (0.95-1.53)	.12	NA	NA	1.17 (0.92-1.48)	.21	NA	NA

Abbreviations: HR, hazard ratio; NA, not applicable; PRS, polygenic risk score; SNV, single nucleotide variant; TC, thyroid cancer.

^a^ Model 1 was not adjusted; model 2 was adjusted for age, sex, genetic composition, Townsend deprivation index at recruitment, educational qualifications, and average total household income before tax.

^b^ PRSs were determined as continuous variables using Cox proportional hazards models.

^c^ PRS1 (15 SNVs) originated from the meta-GWAS analysis (*P* < 5 × 10^−5^).

### Association Between Modifiable Lifestyles and TC

As reported in Table 3, after adjustment for covariates in model 2, adherence to an unfavorable lifestyle was associated with TC risk (intermediate vs favorable HR, 1.54; 95% CI, 1.20-1.98; unfavorable vs favorable HR, 1.93; 95% CI, 1.50-2.49; *P* < .001 for trend) in contrast to those with a favorable lifestyle. Patients with unfavorable moderate to vigorous physical activity (HR, 1.41; 95% CI, 1.17-1.72; *P* < .001), weight (HR, 1.33; 95% CI, 1.09-1.63; *P* = .01), and smoke intake (HR, 1.62; 95% CI, 1.18-2.23; *P* = .003) would be more likely to have a higher risk of TC; however, unfavorable alcohol consumption presented a reverse trend (HR, 0.70; 95% CI, 0.55-0.88; *P* = .002). We did not find an association between diet index and TC (HR, 1.04; 95% CI, 0.85-1.26; *P* = .72). Furthermore, we compared the difference between genetic risk, lifestyle, and different histologic types and found no significant differences between patients with follicular thyroid cancer and papillary thyroid cancer (eTable 9 in the Supplement).

**Table 3.** Associations Between Healthy Lifestyle Component and Incident Thyroid Cancer

Characteristic	Model 1^a^			Model 2^a^		
	HR (95% CI)	*P* value	*P* value for trend^b^	HR (95% CI)	*P* value	*P* value for trend^b^
Weighted healthy lifestyle						
Favorable	1 [Reference]	NA	<.001	1 [Reference]	NA	<.001
Intermediate	1.79 (1.39-2.30)	<.001		1.54 (1.20-1.98)	<.001	
Unfavorable	1.76 (1.37-2.26)	<.001		1.93 (1.50-2.49)	<.001	
Unweighted healthy lifestyle						
Favorable	1 [Reference]	NA	.11	1 [Reference]	NA	.01
Intermediate	1.13 (0.90-1.43)	.29		1.12 (0.89-1.42)	.33	
Unfavorable	1.26 (1.00-1.59)	.05		1.40 (1.10-1.78)	.01	
Total fruit and vegetable intake						
Favorable	1 [Reference]	NA	.23	1 [Reference]	NA	>.99
Unfavorable	0.86 (0.69-1.05)	.14		0.94 (0.76-1.16)	.58	
Fish intake						
Favorable	1 [Reference]	NA	.46	1 [Reference]	NA	.70
Unfavorable	0.90 (0.72-1.14)	.39		0.94 (0.74-1.19)	.60	
Processed meat and red meat intake						
Favorable	1 [Reference]	NA	.02	1 [Reference]	NA	.82
Unfavorable	0.80 (0.65-0.97)	.03		1.00 (0.81-1.24)	>.99	
Whole grains intake						
Favorable	1 [Reference]	NA	.65	1 [Reference]	NA	.17
Unfavorable	0.93 (0.76-1.14)	.49		0.94 (0.76-1.15)	.54	
Refined grains intake						
Favorable	1 [Reference]	NA	.07	1 [Reference]	NA	.90
Unfavorable	0.88 (0.73-1.06)	.18		0.97 (0.80-1.18)	.75	
Sugar drinking intake						
Favorable	1 [Reference]	NA	NA	1 [Reference]	NA	NA
Unfavorable	0.99 (0.76-1.27)	.91		1.14 (0.88-1.49)	.33	
Diet index						
Favorable	1 [Reference]	NA	.03	1 [Reference]	NA	.69
Unfavorable	0.90 (0.74-1.09)	.27		1.04 (0.85-1.26)	.72	
Total moderate to vigorous physical activity						
Favorable	1 [Reference]	NA	NA	1 [Reference]	NA	NA
Unfavorable	1.46 (1.20-1.77)	<.001		1.41 (1.17-1.72)	<.001	
Smoke intake						
Favorable	1 [Reference]	NA	NA	1 [Reference]	NA	NA
Unfavorable	1.44 (1.06-1.95)	.02		1.62 (1.18-2.23)	.003	
Alcohol consumption						
Favorable	1 [Reference]	NA	.01	1 [Reference]	NA	.02
Unfavorable	0.63 (0.50-0.79)	<.001		0.70 (0.55-0.88)	.002	
Healthy weight						
Favorable	1 [Reference]	NA	<.001	1 [Reference]	NA	<.001
Unfavorable	1.39 (1.14-1.70)	.001		1.33 (1.09-1.63)	.01	

Abbreviations: HR, hazard ratio; NA, not applicable.

^a^ Model 1 was not adjusted; model 2 was adjusted for age, sex, genetic composition, Townsend deprivation index at recruitment, educational qualifications, and average total household income before tax.

^b^ *P* values for trend were determined by using lifestyle factors as a continuous variable.

### Interaction Association of Lifestyle, Genetic Factors, and TC

Table 4 displays the association between lifestyle and TC in the PRS-stratified analysis with unfavorable lifestyle as the reference. We observed that unfavorable lifestyle was associated with TC in the higher PRS group (favorable vs unfavorable HR, 0.52; 95% CI, 0.37-0.73; *P* < .001). Similar results were shown with unweighted lifestyle and smoking. However, no significant multiplicative interactions were observed. The additive association is presented in eTable 10 in the Supplement, with a favorable lifestyle and lower PRS as the reference. A positive additive association was identified only in patients with high PRSs and an unfavorable lifestyle (RERI: 1.90; 95% CI, 0.44-3.35) and smoking (RERI: 2.79; 95% CI, 0.45-5.12). Intermediate PRSs and lifestyle also presented a positive additive association with TC (RERI: 1.16; 95% CI, 0.20-2.12). The results did not materially change in the nested case-control design (eTable 11 in the Supplement).

**Table 4.** Associations of Lifestyle Component With Incident Thyroid Cancer According to PRS Stratified Analysis^a^

Characteristic	PRS1		PRS2		PRS3		*P* value for interaction
	HR (95% CI)	*P* value	HR (95% CI)	*P* value	HR (95% CI)	*P* value	
Weighted healthy lifestyle							
Unfavorable	1 [Reference]	NA	1 [Reference]	NA	1 [Reference]	NA	.43
Intermediate	0.88 (0.50-1.54)	.65	1.00 (0.67-1.49)	.98	0.66 (0.48-0.92)	.01	
Favorable	0.70 (0.39-1.26)	.23	0.42 (0.25-0.70)	<.001	0.52 (0.37-0.73)	<.001	
Unweighted healthy lifestyle							
Unfavorable	1 [Reference]	NA	1 [Reference]	NA	1 [Reference]	NA	.48
Intermediate	0.52 (0.29-0.94)	.03	1.13 (0.73-1.74)	.57	0.75 (0.54-1.06)	.10	
Favorable	0.67 (0.39-1.14)	.14	0.78 (0.50-1.24)	.29	0.71 (0.51-0.99)	.046	
Diet index							.47
Unfavorable	1 [Reference]	NA	1 [Reference]	NA	1 [Reference]	NA	
Favorable	0.67 (0.42-1.07)	.09	1.05 (0.73-1.50)	.81	1.10 (0.83-1.46)	.50	
Total moderate to vigorous physical activity							
Unfavorable	1 [Reference]	NA	1 [Reference]	NA	1 [Reference]	NA	.90
Favorable	0.73 (0.46-1.16)	.19	0.65 (0.46-0.92)	.02	0.74 (0.57-0.97)	.03	
Smoke intake							
Unfavorable	1 [Reference]	NA	1 [Reference]	NA	1 [Reference]	NA	.16
Favorable	0.88 (0.37-2.08)	.77	0.73 (0.39-1.38)	.34	0.48 (0.32-0.72)	<.001	
Alcohol consumption							
Unfavorable	1 [Reference]	NA	1 [Reference]	NA	1 [Reference]	NA	.27
Favorable	1.07 (0.64-1.79)	.80	1.88 (1.18-2.99)	.01	1.36 (0.99-1.86)	.06	
Healthy weight							
Unfavorable	1 [Reference]	NA	1 [Reference]	NA	1 [Reference]	NA	.33
Favorable	0.93 (0.58-1.48)	.75	0.67 (0.46-0.97)	.03	0.76 (0.58-1.01)	.06	

Abbreviations: HR, hazard ratio; NA, not applicable; PRS, polygenic risk score.

^a^ Adjusted for age, sex, genetic composition, Townsend deprivation index at recruitment, educational qualifications, and average total household income before tax.

### Combined Association of Lifestyle, Genetic Factors, and TC

The combined analysis of the PRS and lifestyle factors and the risk of TC is presented in the Figure. We observed a monotonic association between increasing PRSs and unfavorable lifestyle with a higher risk of TC, and participants with the highest PRS and an unfavorable lifestyle had the highest risk of TC (HR, 4.89; 95% CI, 3.03-7.91; *P* < .001). A similar pattern was observed for the unweighted lifestyle (eTable 12 in the Supplement). In addition, we integrated the PRS and 5 lifestyle behaviors to explore their association with the risk of TC, with the lowest PRS and favorable lifestyle component as the reference group (eTable 12 in the Supplement). The increased risk of TC was related to 2 unfavorable lifestyle behaviors (moderate to vigorous physical activity and weight) across the strata of the PRS. The pattern of smoking was consistent only in the intermediate and high PRS groups. There was no combined association between alcohol consumption, PRS, and TC.

**Figure.**
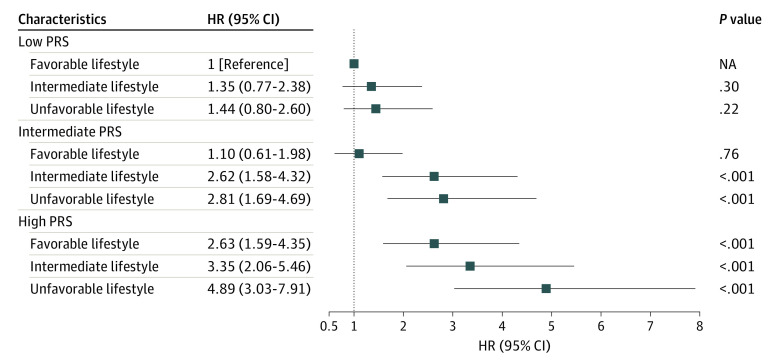
Joint Association Between Polygenic Risk Score (PRS), Lifestyle, and Thyroid Cancer HR indicates hazard ratio; NA, not applicable.

## Discussion

In our analysis, participants who had a higher PRS and did not adhere to healthy lifestyle recommendations were independently associated with an increased risk of TC. In addition, adherence to a healthier lifestyle could decrease the incidence of TC in individuals with a higher PRS. Furthermore, participants with both an unfavorable lifestyle and a higher PRS had the highest risk of TC.

In this study, participants with a higher PRS were significantly more susceptible to TC, which is consistent with previous studies.^6,30^ Previous GWASs had confirmed these genetic variants related to TC. Our meta-GWAS analysis also validated reported genes, such as *NRG1*, *DIRC3*, *FOX1*, *EPB41L4A*, and *LOC105370452*. We achieved more than 80% power using PRS1 with an odds ratio greater than 1.30. In addition, according to previous studies of PRS and GWAS analysis, we constructed several PRS models derived from different approaches and compared their differences through performance evaluation and the number of SNVs. Better performance and a small number of SNVs were noted with PRS1 (eFigure 3 in the Supplement). Therefore, we considered the 15 SNV PRSs in the subsequent analysis. Furthermore, the results did not substantially change using other PRSs (eTable 8 in the Supplement).

Our findings suggest that an unfavorable lifestyle was associated with TC. To our knowledge, this is the first study to combine 5 factors into 1 variable to explore the association between TC and lifestyle. Previous studies only evaluated the role of single lifestyle factors and did not consider that lifestyle behaviors often coexist. According to the World Cancer Research Fund and American Institute for Cancer Research recommendations, adherence to a healthy lifestyle is related to a lower risk of cancer incidence, which has been validated in breast cancer and other cancer types.^31,32^ Furthermore, we noted the positive association of weight and the negative association of alcohol use^33,34,35^ with TC risk, which is in accord with most earlier observations and experiments.^33,36^ The possible mechanisms behind the benefits of alcohol use may be associated with suppression of thyroid hormone metabolism,^37^ induction of thyroid gland dysfunction,^37,38^ and the function of the hypothalamic-pituitary-thyroid axis.^39^ In addition, we noted a nonlinear association between alcohol consumption and TC using adjusted restricted cubic spline (eFigure 4 in the Supplement). The restricted cubic spline plot showed a nonlinear association between TC among men and women: women had more than 2.1 drink-equivalents and men had less than 1.2 drink-equivalents with an HR greater than 1. Therefore, the association between alcohol consumption and TC needs to be validated in more studies. There were positive associations between moderate to vigorous physical activity and TC, which was also reported by Xhaard et al.^35^ The mechanism is still unclear, but may partly be explained by regular exercise reducing the occurrence of various cancers by maintaining normal levels of sex hormones, insulin, and leptin.^7,14,40,41^ Null significance was found between the dietary index and TC. Whether dietary factors were related to TC remain debatable.^42,43^ This discrepancy might be attributed to the amount of iodized salt in food.^44,45^ We found a positive association between smoking and TC, which was controversial in previous studies. Smoking was shown to be protective against TC in some studies,^38,46^ and other studies have reported null significance.^47,48,49^ Multiple studies^50,51,52^ have noted that current smoking was associated with reduced thyroid-stimulating hormone levels, resulting in the decreased stimulation of the thyroid gland. In our study, the stratification analysis showed no substantial link among women—only in men (eTables 13-15 in the Supplement). The sensitivity analysis and competing risk analysis did not validate the results of smoking (eTables 16-20 in the Supplement). Therefore, the inconsistency between smoking and TC may have been influenced by sex and the lack of adequate sample size (only 46 current smokers in the present study). The results need to be interpreted with caution, and further studies are needed.

We first combined lifestyle and PRSs to explore the joint and interactive association with TC and discovered that a healthier lifestyle could diminish the risk of TC in individuals with high PRSs. This finding was in accord with a study^53^ in the UK Biobank that has revealed that genetic and lifestyle factors had a joint association with the risk of overall cancer. Other cancer studies^20,21^ also showed analogous results. In addition, a Korean cancer-screened cohort^54^ illustrated that body mass index and PRS have a cumulative influence on TC. The possible hypothesis between a joint association between genetics, lifestyle factors, and TC may influence thyroid hormone metabolism.^55^ Several GWAS analyses^55,56^ related to thyroid hormones have reported 2 common variants (rs965513 and rs966423), which were also associated with TC. Lifestyle behaviors (weight loss and smoking) may affect the levels of thyroid hormones.^57,58^ L-Thyroxine can stimulate cancer cell proliferation, cancer-relevant angiogenesis, and platelet coagulation.^59^ Therefore, lifestyle and genetic factors may have a joint influence on TC by controlling the changes in thyroid hormones. In addition, the interaction between a high PRS and an unfavorable lifestyle showed no significance at an additive and multiplicative scale, which might partly be due to the relatively small sample size.

To our knowledge, this is the first study to explore the association between lifestyle, genetic factors, and TC risk. Our research conducted a series of sensitivity analyses, including 4 PRSs, stratification analysis of sex, and a nested case-control study. We also conducted a competing risk analysis to support the results, using Cox proportional hazards models.

### Limitations

Our study also has limitations. First, because the lifestyle data of only 502 505 individuals were available at baseline, we could not measure longitudinal changes in lifestyle. We attempted to reduce the influence of lifestyle changes by excluding patients with a history of cancer. Second, there is an absence of relevant data about iodine intake, radiation exposure, experience, and family history. Third, due to the inadequate number of pathologic types of TC, it was hard to explore the association between lifestyle and genetics in different pathologic types of TC. We also classified TC into different histologic types. Of 423 TC cases, there were only 370 cases that had specified histologic types (follicular TC = 55, papillary TC = 207, follicular and papillary TC = 88, anaplastic TC = 9, and medullar TC = 11), and 99 cases had unspecified histologic types. There was no significant difference between patients with FTC and PTC (eTable 9 in the Supplement). Fourth, compared with the genetic predisposition (8%) in the previous study using 10 SNV PRSs,^6^ we only noted a similar genetic predisposition (5%), which may be due to the small sample size of TC cases in the meta-GWAS analysis. However, we constructed several PRSs and validated their stability. Fifth, the association between an unfavorable lifestyle, PRS, and TC was observed only in individuals of European descent; thus, the results of this study need to be generalized with caution to other populations.

## Conclusions

The findings of this study suggest that adherence to a healthier lifestyle could attenuate the deleterious role of genetic factors on the risk of TC, especially in individuals at a high genetic risk. Hence, lifestyle interventions may be beneficial for preventing TC, especially in individuals with a high genetic predisposition.

## Notes

Supplement. **eMethods 1.** GWAS Analysis **eMethods 2.** The Procedure of Constructing Other PRSs **eAppendix.** The Result of GWAS Analysis **eTable 1.** Healthy Lifestyle Components **eTable 2.** SNPs Associated With Thyroid Cancer From PubMed, Ensemble, and MR-Base Platform **eTable 3.** Baseline Characteristics of Six Diet Factors in the UK Biobank **eTable 4.** Baseline Characteristics Between Women and Men **eTable 5.** GWAS Results of UK Biobank (*P* < 5 × 10^−8^) **eTable 6.** Meta-GWAS Results of Three Cohorts (n = 1956) **eTable 7.** Genetic Risk Score Selection **eTable 8.** Association of Different PRSs and Risk of Thyroid Cancer **eTable 9.** Baseline Characteristics of Thyroid Cancer Participants Between FTC and PTC **eTable 10.** The Additive Interaction Between Lifestyle and PRS (RERI) **eTable 11.** Associations of Lifestyle Components With Incident Thyroid Cancer According to PRS Stratified Analysis in the Nested Case-Control Design **eTable 12.** Combined Analysis of PRS and Lifestyle Components on the Risk of Thyroid Cancer **eTable 13.** The Sex Difference of Associations Between Lifestyle Components With Incident Thyroid Cancer **eTable 14.** Combined Analysis of PRS and Lifestyle Components on the Risk of Thyroid Cancer in Women **eTable 15.** Combined Analysis of PRS and Lifestyle Components on the Risk of Thyroid Cancer in Men **eTable 16.** Baseline Characteristics of Participants of Thyroid Cancer in the UK Biobank in the Sensitivity Analysis **eTable 17.** Associations Between Healthy Lifestyle and Incident Thyroid Cancer in the Sensitivity Analysis **eTable 18.** Combined Analysis of PRS and Lifestyle Components on the Risk of Thyroid Cancer in the Sensitivity Analysis **eTable 19.** Associations Between Lifestyle Component and Incident Thyroid Cancer Using Competing Risk Analysis **eTable 20.** Combined Analysis of PRS and Lifestyle Components on the Risk of Thyroid Cancer Using Competing Risk Analysis **eFigure 1.** Flow Diagram for the Selection of TC Cases in the UK Biobank **eFigure 2.** Manhattan Plot and QQ Plot of Meta-GWAS of Thyroid Cancer **eFigure 3.** ROC Curve of Genetic Risk Score **eFigure 4.** Restricted Cubic Spline Curve of Drink Score
eReferences
