# Supplementary material for: Association Between Genetic Risk, Adherence to Healthy Lifestyle Behavior, and Thyroid Cancer Risk
Source: JAMA Netw Open. 2022 Dec 12;5(12):e2246311. doi: 10.1001/jamanetworkopen.2022.46311 (PMC9856466; doi:10.1001/jamanetworkopen.2022.46311)
Supplement: Supplement. — eMethods 1. GWAS Analysis eMethods 2. The Procedure of Constructing Other PRSs eAppendix. The Result of GWAS Analysis eTable 1. Healthy Lifestyle Components eTable 2. SNPs Associated With Thyroid Cancer From PubMed, Ensemble, and MR-Base Platform eTable 3. Baseline Characteristics of Six Diet Factors in the UK Biobank eTable 4. Baseline Characteristics Between Women and Men eTable 5. GWAS Results of UK Biobank (P < 5 × 10^−8^) eTable 6. Meta-GWAS Results of Three Cohorts (n = 1956) eTable 7. Genetic Risk Score Selection eTable 8. Association of Different PRSs and Risk of Thyroid Cancer eTable 9. Baseline Characteristics of Thyroid Cancer Participants Between FTC and PTC eTable 10. The Additive Interaction Between Lifestyle and PRS (RERI) eTable 11. Associations of Lifestyle Components With Incident Thyroid Cancer According to PRS Stratified Analysis in the Nested Case-Control Design eTable 12. Combined Analysis of PRS and Lifestyle Components on the Risk of Thyroid Cancer eTable 13. The Sex Difference of Associations Between Lifestyle Components With Incident Thyroid Cancer eTable 14. Combined Analysis of PRS and Lifestyle Components on the Risk of Thyroid Cancer in Women eTable 15. Combined Analysis of PRS and Lifestyle Components on the Risk of Thyroid Cancer in Men eTable 16. Baseline Characteristics of Participants of Thyroid Cancer in the UK Biobank in the Sensitivity Analysis eTable 17. Associations Between Healthy Lifestyle and Incident Thyroid Cancer in the Sensitivity Analysis eTable 18. Combined Analysis of PRS and Lifestyle Components on the Risk of Thyroid Cancer in the Sensitivity Analysis eTable 19. Associations Between Lifestyle Component and Incident Thyroid Cancer Using Competing Risk Analysis eTable 20. Combined Analysis of PRS and Lifestyle Components on the Risk of Thyroid Cancer Using Competing Risk Analysis eFigure 1. Flow Diagram for the Selection of TC Cases in the UK Biobank eFigure 2. Manhattan Plot and QQ Plot of Meta-GWAS of Thyroid Cancer [file jamanetwopen-e2246311-s001.pdf]

Supplementary Online Content

Feng X, Wang F, Yang W, et al. Association between genetic risk, adherence to healthy lifestyle behavior, and thyroid cancer risk. *JAMA Netw Open*. 2022;5(12):e2246311. doi:10.1001/jamanetworkopen.2022.46311

eMethods 1. GWAS Analysis

eMethods 2. The Procedure of Constructing Other PRSs

eAppendix. The Result of GWAS Analysis

eTable 1. Healthy Lifestyle Components

eTable 2. SNPs Associated With Thyroid Cancer From PubMed, Ensemble, and MR-Base Platform

eTable 3. Baseline Characteristics of Six Diet Factors in the UK Biobank

eTable 4. Baseline Characteristics Between Women and Men

eTable 5. GWAS Results of UK Biobank ($P < 5 \times 10^{-8}$)

eTable 6. Meta-GWAS Results of Three Cohorts (n=1956)

eTable 7. Genetic Risk Score Selection

eTable 8. Association of Different PRSs and Risk of Thyroid Cancer

eTable 9. Baseline Characteristics of Thyroid Cancer Participants Between FTC and PTC

eTable 10. The Additive Interaction Between Lifestyle and PRS (RERI)

eTable 11. Associations of Lifestyle Components With Incident Thyroid Cancer According to PRS Stratified Analysis in the Nested Case-Control Design

eTable 12. Combined Analysis of PRS and Lifestyle Components on the Risk of Thyroid Cancer

eTable 13. The Sex Difference of Associations Between Lifestyle Components With Incident Thyroid Cancer

eTable 14. Combined Analysis of PRS and Lifestyle Components on the Risk of Thyroid Cancer in Women

eTable 15. Combined Analysis of PRS and Lifestyle Components on the Risk of Thyroid Cancer in Men

eTable 16. Baseline Characteristics of Participants of Thyroid Cancer in the UK Biobank in the Sensitivity Analysis

eTable 17. Associations Between Healthy Lifestyle and Incident Thyroid Cancer in the Sensitivity Analysis

eTable 18. Combined Analysis of PRS and Lifestyle Components on the Risk of Thyroid Cancer in the Sensitivity Analysis

eTable 19. Associations Between Lifestyle Component and Incident Thyroid Cancer Using Competing Risk Analysis

eTable 20. Combined Analysis of PRS and Lifestyle Components on the Risk of Thyroid Cancer Using Competing Risk Analysis

eFigure 1. Flow Diagram for the Selection of TC Cases in the UK Biobank

eFigure 2. Manhattan Plot and QQ Plot of Meta-GWAS of Thyroid Cancer

eFigure 3. ROC Curve of Genetic Risk Score

eFigure 4. Restricted Cubic Spline Curve of Drink Score

eReferences

This supplementary material has been provided by the authors to give readers additional information about their work.

© 2022 Feng X et al. *JAMA Network Open.*

eMethods 1: GWAS analysis

The first stage of GWAS was divided into two steps: performed GWAS on the participants of UK Biobank and further conducted meta-GWAS using three cohorts (including UK Biobank). The GWAS of UK Biobank was performed on all TC patients and 1,031 TC cases by the newly released data (July, 2021). Then we match 5,155 controls without any cancers according to age (± 5 years old), sex, race, assessment center and year of enrollment, which were included in the next GWAS. Samples were genotyped using the UK Biobank Axiom array and UK BiLEVE Axiom Array. The released genotype data were imputed with the reference to the Haplotype Reference Consortium panel. Detailed genotyping and quality control (QC) procedures were described on the UK Biobank website (http://biobank.ctsu.ox.ac.uk/). Downstream QC procedures conducted for the next analysis: (1) samples or variants with call rate < 0.99, (2) individuals with kinship coefficient > 0.0884, (3) genotypic and phenotypic sex mismatch, (4) heterozygosity rate deviating more than three standard deviations from the mean and missingness of phenotypes or covariates, (5) missingness of phenotypes or covariates, (6) variants with minor allele frequency (MAF) < 0.01, Hardy-Weinberg equilibrium $P_{(PHWE)} <1 .0 \times 10^{-6}$. After removing these samples and variants, we retained 6,121 samples (986 cases and 5,135 controls) and 6,562,992 SNPs for subsequent GWAS analysis. Then we conducted a fixed-effect Meta GWAS using summary data of UK Biobank (986 cases and 5,135 controls), the FinnGen Study including 321 TC and 96,178 controls and Italian residents based on 649 differentiated TC cases and 431 controls. (1) Hence, a total of 1956 TC cases and 101,924 controls were included in this meta-analysis. Relevant GWAS summary data could be found on the website (https://gwas.mrcieu.ac.uk/datasets/ieu-a-1082/; https://gwas.mrcieu.ac.uk/datasets/finn-a-C3_THYROID_GLAND/). Adjusted for age, BMI, year of enrollment, assessment center and the first five ancestry principal components (PCs). GWAS Manhattan

© 2022 Feng X et al. *JAMA Network Open.*

and Quantile-Quantile (QQ) plots were produced and checked for each variant. The genomic inflation factor was calculated by linkage disequilibrium score regression analysis (LDSC).

© 2022 Feng X et al. *JAMA Network Open.*

eMethods 2 The procedure of constructing other PRSs

Through searching GWAS-related studies of TC in PubMed, Ensemble, and MR-base platform, we included eight GWAS research and one PRS research of European related to TC (Supplementary table S2). Finally, four PRS were calculated: the PRS (PRS1) deprived of our Meta-GWAS analysis with $P<5\times10^{-5}$, three secondary PRS deprived of our Meta-GWAS analysis with $P < 5\times10^{-6}$ (PRS2), published eight GWAS analyses (PRS3) and the PRS research of European (PRS4), respectively [1-9]. All PRS were abstracted from autosome with $r^2 < 0.99$, MAF ≥ 0.01 and $P < 5\times10^{-5}$. And we evaluated and selected the better PRS through the predictive power and the strength of association.

© 2022 Feng X et al. *JAMA Network Open.*

eAppendix. The result of GWAS analysis

In the GWAS of UK Biobank, we identified 223 genetic loci associated with TC risk of $P<5\times10^{-8}$ on chromosomes 2 and 9, which were in extremely strong LD with rs965513 on chromosome 9 and rs6759952 on chromosome 2 (known SNP related with TC, $r^2 < 0.99$). The GWAS result was presented in Table S3, with the threshold $P < 5\times10^{-8}$. For replication, we conducted a GWAS meta-analysis using GWAS summary statistics from UK Biobank, the FinnGen Study and Italian residents. This meta-analysis was conducted using a fixed-effect inverse variance weighted method, and significant SNPs in three cohorts with $P<5\times10^{-5}$ and Q (the Cochran's Q-test) > 0.05 were considered. There were 41 SNPs meeting the criteria, and two top SNPs (rs965513, *FOXE1* of chromosome 9, $P_{\text{meta}} = 5.60\times10^{-40}$); rs6759952, *DIRC3* of chromosome 2, $P_{\text{meta}} = 4.36\times10^{-11}$) independently associated with TC ($r^2 < 0.99$). Other SNPs with $P > 5\times10^{-8}$ and $P < 5\times10^{-5}$ were listed in Table S4. Manhattan and QQ plots are in eFigure 1, and the genomic inflation factor (λ) is 1.037.

© 2022 Feng X et al. *JAMA Network Open.*

eTable 1 Healthy lifestyle components		
Healthy lifestyle factors	Source and definition	Self-reported UK Biobank field code
1. Smoke	Never and previous	Favourable
	Current	Unfavourable
2. Total moderate-vigorous physical activity (min/wk):	>=150 minutes moderate activity per week or >= 75 minutes vigorous activity per week	Favourable
	Other	Unfavourable
3. Diet index	UK Biobank Food Frequency Questionnaire at baseline;	
	At least 4 of the following 7 food groups:	
	1. Fruits and Vegetables: >= 5 servings/day	
	2. Fish: >=3 servings/week	
	3. red meat Processed meats <=4 servings/week	
	4. Whole grains: >=5.5 servings/day	
	5. Refined grains <=2 servings/week	
	6. Sugar-sweetened beverages =0 servings/week	
	Adequate intake of >=3 dietary components	Favourable
	Adequate intake of <3 dietary components	Unfavourable
4. Moderate alcohol consumption	UK Biobank Touchscreen questionnaire at baseline; US Dietary guidelines for Americans 2015-2020 of up to 1 drink/day for women and up to 2 drinks/day for men.	
	Women: <14g/day 1 drink-equivalent	Favourable
	Men: <28g/day 2 drink-equivalent	
	Women>=14 1 drink-equivalent	Unfavourable
	Men>=28 2 drink-equivalent	
5. Be a Weight	BMI (kg/m^2)	
	18.5-24.9	Favourable
	25-29.9	Intermediate
	>=30 or <18.5	Unfavourable
	Waist circumference (cm(in))	
	Men: <94 (<37)	Favourable
	Women: <80 (<31.5)	
	Men: 94-<102 (37-<40)	Intermediate

© 2022 Feng X et al. *JAMA Network Open.*

	Women: 80-<88 (31.5-<35)	
	Men: >=102 (>=40)	Unfavourable
	Women: >=88 (>=35)	
	Weight	
	The sum score of BMI and waist circumference >=4	Favourable
	The sum score of BMI and waist circumference < 4	Unfavourable
Lifestyle	3-5 favorable lifestyle factors	Favourable
	2 favorable lifestyle factors	Intermediate
	0-1 favorable lifestyle factors	Unfavourable

eTable 2 SNPs associated with thyroid cancer from PubMed, Ensemble, and MR-base platform

CHR	Position	SNP	MAF	Gene	*P*	PubMed ID
9	97793827	rs965513	0.34	*FOXE1*	2.00E-27	19198613
14	36180040	rs944289	0.42	*NKX2-1*	2.00E-09	19198613
14	36269155	rs116909374	0.04	*MBIP*	5.00E-11	22267200
2	217445617	rs966423	0.43	*DIRC3*	1.00E-09	22267200
8	32574851	rs2439302	0.47	*NRG1*	2.00E-09	22267200
9	97793827	rs965513	0.34	*FOXE1*	3.00E-10	23894154
2	217406996	rs6759952	0.44	*DIRC3*	6.00E-10	23894154
5	1279675	rs10069690	0.28	*TERT*	3.20E-07	23894154
7	110540965	rs10238549	0.32	*LOC105375451*	4.10E-06	23894154
9	136374886	rs10781500	0.39	*CARD9*	3.50E-05	23894154
3	158745312	rs7617304	0.22	*LOC100287290*	4.60E-05	23894154
20	39318791	rs7267944	0.19	*DHX35*	1.34E-08	25029422
9	97793827	rs965513	0.34	*FOXE1*	3.00E-23	25855579
9	97896036	rs7037324	0.22	*FOXE1*	1.00E-17	25855579
9	97865986	rs10122541	0.37	*FOXE1*	1.00E-17	25855579
9	97896036	rs7037324	0.37	*FOXE1*	1.00E-17	25855579
9	97775520	rs1588635	0.34	*PTCSC2*	2.00E-58	28195142
14	36269155	rs116909374	0.04	*MBIP*	1.00E-16	28195142
15	67163292	rs56062135	0.21	*SMAD3*	5.00E-09	28195142
5	1279675	rs10069690	0.28	*TERT*	3.00E-07	28195142
9	97772985	rs7030280	0.34	*PTCSC2*	5.00E-28	32887889
8	32575620	rs2466074	0.47	*NRG1*	5.00E-11	32887889
9	97886005	rs12002967	0.11	*FANCC*	3.00E-07	32887889
1	218449752	rs6697759	0.45	*RRP15*	4.00E-07	32887889
10	72033486	rs72806259	0.1	*Intergenic*	5.00E-07	32887889
4	112649292	rs76032629	0.03	*LARP7, MIR302A, MIR302B , MIR302C, MIR302D, MIR367, MIR302CHG*	6.00E-07	32887889
4	156130312	rs555678255	0.1	*LOC102724785*	8.00E-07	32887889
11	20338417	rs74518511	0.03	*Intergenic*	9.00E-07	32887889
15	97673727	rs77166399	0.12	*LINC00923*	9.00E-07	32887889
9	97775520	rs1588635	0.34	*NR*	2.00E-58	33527407
16	79668474	rs16950982	0.35	*MAF*	4.70E-09	33527407
1	61142801	rs334729	0.04	*NFIA*	8.70E-08	33527407
19	22065515	rs10415826	0.2	*ZNF257*	8.70E-07	33527407

© 2022 Feng X et al. *JAMA Network Open.*

2	217427435	rs11693806	0.29	*DIRC3*	1.50E-24	32132206 *
14	36063370	rs368187	0.46	*LOC105370452*	5.10E-23	32132206 *
8	32575278	rs2466076	0.47	*NRG1*	1.50E-17	32132206 *
1	233276815	rs12129938	0.22	*PCNX2*	4.00E-11	32132206 *
10	103934543	rs7902587	0.09	*OBFC1*	5.40E-11	32132206 *
5	112150207	rs73227498	0.13	*EPB41L4A*	3.00E-10	32132206 *
15	67165147	rs2289261	0.37	*SMAD3*	3.10E-09	32132206 *
3	169800667	rs6793295	0.27	*LRRC34*	2.70E-08	32132206 *

CHR=chromosome; SNP=single nucleotide polymorphisms; MAF=minor allele frequency.

Note: the published literature (PMID: 32132206) was research on the relationship between polygenic risk score and thyroid cancer

© 2022 Feng X et al. *JAMA Network Open.*

eTable 3 Baseline Characteristics of six diet factors in the UK Biobank

Variable[a]	Overall (n=264,956)	Non incident TC (n= 264,533)	Incident TC (n=423)	*P*
Total fruit and vegetables intake				
Unfavourable	196312 (74.09)	196012 (74.10)	300 (70.92)	0.15
Favourable	68644 (25.91)	68521 (25.90)	123 (29.08)	
Fish intake				
Unfavourable	64922 (24.50)	64826 (24.51)	96 (22.70)	0.42
Favourable	200034 (75.50)	199707 (75.49)	327 (77.30)	
Processed meat and red meat intake				
Unfavourable	190665 (71.96)	190381 (71.97)	284 (67.14)	0.03
Favourable	74291 (28.04)	74152 (28.03)	139 (32.86)	
Whole grains intake				
Unfavourable	93786 (35.40)	93643 (35.40)	143 (33.81)	0.53
Favourable	171170 (64.60)	170890 (64.60)	280 (66.19)	
Refine grains intake				
Unfavourable	137401 (51.86)	137195 (51.86)	206 (48.70)	0.21
Favourable	127555 (48.14)	127338 (48.14)	217 (51.30)	
Sugar drinking intake				
Unfavourable	221086 (83.44)	220734 (83.44)	352 (83.22)	0.95
Favourable	43870 (16.56)	43799 (16.56)	71 (16.78)	
[a] Variables are numbers (percentages)				

© 2022 Feng X et al. *JAMA Network Open.*

eTable 4 Baseline characteristics between women and men

Variable[a]	Women (n=137,665)	Men (n=127,291)	*P*
Thyroid			<0.001
Case	302 (71.39)	121 (28.61)	
Control	137363 (99.78)	127170 (99.90)	
Age	57 (49, 62)	57 (49, 63)	0.003
Townsend deprivation index			<0.001
1 (least deprived)	30684 (22.29)	28219 (22.17)	
2-4	86362 (62.73)	78295 (61.51)	
5 (most deprived)	20619 (14.98)	20777 (16.32)	
Educational qualifications			<0.001
College or University degree	45640 (33.15)	45753 (35.94)	
Secondary Education	79005 (57.39)	64471 (50.65)	
Some professional qualifications	13020 (9.46)	17067 (13.41)	
Average total household income before tax			<0.001
Less than 18,000	24359 (17.73)	19451 (15.33)	
18,000 to 30,999	30605 (22.28)	27478 (21.65)	
31,000 to 51,999	51009 (37.13)	43099 (33.96)	
52,000 to 100,000	25000 (18.20)	29078 (22.91)	
Greater than 100,000	6398 (4.66)	7794 (6.14)	
Total fruit and vegetables intake			
Unfavourable	96592 (70.16)	99720 (78.34)	<0.001
Favourable	41073 (29.84)	27571 (21.66)	
Fish intake			
Unfavourable	31767 (23.08)	33155 (26.05)	<0.001
Favourable	105898 (76.92)	94136 (73.95)	
Processed meat and red meat intake			
Unfavourable	88013 (63.93)	102652 (80.64)	<0.001
Favourable	49652 (36.07)	24639 (19.36)	
Whole grains intake			
Unfavourable	43310 (31.46)	50476 (39.65)	<0.001
Favourable	94355 (68.54)	76815 (60.35)	
Refine grains intake			
Unfavourable	61852 (44.93)	75549 (59.35)	<0.001
Favourable	75813 (55.07)	51742 (40.65)	
Sugar drinking intake			
Unfavourable	115839 (84.15)	105247 (82.68)	<0.001
Favourable	21826 (15.85)	22044 (17.32)	
Diet index			<0.001
Unfavourable	53987 (39.22)	68932 (54.15)	
Favourable	83678 (60.78)	58359 (45.85)	
Total moderate-vigorous physical activity			<0.001
Unfavourable	65634 (47.68)	56736 (44.57)	

© 2022 Feng X et al. *JAMA Network Open.*

Favourable	72031 (52.32)	70555 (55.43)	
Weight			<0.001
Unfavourable	72920 (52.97)	73545 (57.78)	
Favourable	64745 (47.03)	53746 (42.22)	
Smoke intake			<0.001
Unfavourable	8333 (6.05)	12424 (9.76)	
Favourable	129332 (93.95)	114867 (90.24)	
Alcohol consumption			<0.001
Unfavourable	41153 (29.89)	40500 (31.82)	
Favourable	96512 (70.11)	86791 (68.18)	
Unweighted lifestyle			<0.001
Unfavourable	33621 (24.42)	41231 (32.39)	
Intermediate	45718 (33.21)	41969 (32.97)	
Favourable	58326 (42.37)	44091 (34.64)	
Weighted lifestyle			
Unfavourable	29989 (21.78)	59046 (46.39)	<0.001
Intermediate	55116 (40.04)	26799 (21.05)	
Favourable	52560 (38.18)	41446 (32.56)	
PRS			
T1	45595 (33.12)	42728 (33.57)	0.002
T2	46295 (33.63)	42019 (33.01)	
T3	45775 (33.25)	42544 (33.42)	

PRS=polygenic risk score; TC=thyroid cancer.

a Variable are numbers (percentages) except for age, which was presented as median and interquartile range

© 2022 Feng X et al. *JAMA Network Open.*

eTable 5 GWAS results of UK Biobank ($P < 5 \times 10^{-8}$)								
CHR	Position	SNP	Effect_allele	Other_allele	BETA	SE	*P*	LD
9	100559011	rs13295081	C	T	0.464	0.059	3.216E-15	LD with rs965513
9	100559114	rs13295254	C	T	0.462	0.059	4.299E-15	LD with rs965513
9	100670272	rs1561957	C	T	0.456	0.059	8.076E-15	LD with rs965513
9	100561486	rs10818090	T	C	0.457	0.059	8.563E-15	LD with rs965513
9	100546600	rs925489	T	C	0.452	0.058	1.049E-14	LD with rs965513
9	100548934	rs7864322	T	C	0.452	0.058	1.091E-14	LD with rs965513
9	100549013	rs7850258	G	A	0.452	0.058	1.091E-14	LD with rs965513
9	100554907	rs4743131	G	C	0.451	0.058	1.166E-14	LD with rs965513
9	100542176	rs10759927	G	A	0.450	0.058	1.289E-14	LD with rs965513
9	100547627	rs4273946	C	G	0.450	0.058	1.298E-14	LD with rs965513
9	100547972	rs7020976	C	T	0.450	0.058	1.321E-14	LD with rs965513
9	100548144	rs7032019	A	G	0.451	0.059	1.322E-14	LD with rs965513
9	100544868	rs7847663	T	C	0.450	0.058	1.339E-14	LD with rs965513
9	100546040	rs4743130	T	C	0.450	0.058	1.339E-14	LD with rs965513
9	100546219	rs1561962	T	C	0.450	0.058	1.339E-14	LD with rs965513
9	100546391	rs925488	A	G	0.450	0.058	1.339E-14	LD with rs965513
9	100537577	rs10983701	A	G	0.450	0.058	1.349E-14	LD with rs965513
9	100537455	rs10983700	C	T	0.450	0.058	1.375E-14	LD with rs965513
9	100538470	rs7028661	G	A	0.450	0.058	1.375E-14	LD with rs965513

© 2022 Feng X et al. *JAMA Network Open.*

9	100540541	rs7021576	T	C	0.450	0.058	1.389E-14	LD with rs965513
9	100541473	rs2401637	C	T	0.450	0.058	1.389E-14	LD with rs965513
9	100550375	rs7030241	A	T	0.450	0.059	1.435E-14	LD with rs965513
9	100550455	rs7027030	C	A	0.450	0.059	1.435E-14	LD with rs965513
9	100539518	rs36052460	AT	A	0.450	0.059	1.467E-14	LD with rs965513
9	100663535	rs111492099	GTTTCC	G	0.450	0.059	1.468E-14	LD with rs965513
9	100538923	rs10818050	G	A	0.449	0.058	1.521E-14	LD with rs965513
9	100550028	rs1443438	C	T	0.449	0.058	1.550E-14	LD with rs965513
9	100556109	rs965513	G	A	0.449	0.059	1.592E-14	
9	100537802	rs1588635	C	A	0.449	0.059	1.774E-14	LD with rs965513
9	100553957	rs10983761	C	A	0.448	0.059	1.881E-14	LD with rs965513
9	100650096	rs7866436	A	G	0.445	0.058	2.004E-14	LD with rs965513
9	100552559	rs10739496	T	C	0.448	0.059	2.026E-14	LD with rs965513
9	100535203	rs7030256	G	C	0.449	0.059	2.091E-14	LD with rs965513
9	100535267	rs7030280	T	C	0.449	0.059	2.091E-14	LD with rs965513
9	100661514	rs113545866	GTGAGGAAGGGAC	G	0.447	0.059	2.200E-14	LD with rs965513
9	100665393	rs7044799	T	A	0.447	0.059	2.200E-14	LD with rs965513
9	100658782	rs758709647	C	CTTAT	0.447	0.058	2.236E-14	LD with rs965513
9	100667599	rs1561961	T	C	0.446	0.058	2.381E-14	LD with rs965513
9	100582024	rs6478413	G	A	0.449	0.059	2.565E-14	LD with rs965513
9	100648780	rs10818214	C	T	0.446	0.059	2.648E-14	LD with rs965513

© 2022 Feng X et al. *JAMA Network Open.*

9	100664474	rs10984253	G	A	0.445	0.058	2.813E-14	LD with rs965513
9	100583195	rs1443432	T	C	0.448	0.059	2.841E-14	LD with rs965513
9	100660824	rs7022148	C	T	0.444	0.058	2.857E-14	LD with rs965513
9	100661100	rs7034336	T	C	0.444	0.058	2.875E-14	LD with rs965513
9	100661367	rs9775370	A	G	0.444	0.058	2.875E-14	LD with rs965513
9	100662379	rs7027221	C	T	0.444	0.058	2.875E-14	LD with rs965513
9	100662396	rs7038998	G	C	0.444	0.058	2.875E-14	LD with rs965513
9	100662671	rs10739526	C	T	0.444	0.058	2.875E-14	LD with rs965513
9	100663700	rs3824495	A	C	0.444	0.058	2.875E-14	LD with rs965513
9	100663745	rs3808893	T	C	0.444	0.058	2.875E-14	LD with rs965513
9	100665669	rs7031386	T	G	0.444	0.058	2.875E-14	LD with rs965513
9	100665698	rs7048255	G	A	0.444	0.058	2.875E-14	LD with rs965513
9	100666543	rs9299258	G	T	0.444	0.058	2.875E-14	LD with rs965513
9	100658938	rs6478469	G	A	0.444	0.058	3.012E-14	LD with rs965513
9	100660145	rs28703314	T	A	0.444	0.058	3.018E-14	LD with rs965513
9	100660896	rs10984235	T	C	0.444	0.058	3.040E-14	LD with rs965513
9	100667871	rs11455506	AT	A	0.445	0.059	3.044E-14	LD with rs965513
9	100656402	rs7032086	G	T	0.444	0.058	3.062E-14	LD with rs965513
9	100660284	rs10984230	T	C	0.444	0.058	3.062E-14	LD with rs965513
9	100661972	rs10818241	G	A	0.444	0.058	3.160E-14	LD with rs965513
9	100652711	rs12006522	C	G	0.443	0.058	3.268E-14	LD with rs965513
9	100664382	rs6478471	G	A	0.443	0.058	3.305E-14	LD with rs965513

© 2022 Feng X et al. *JAMA Network Open.*

9	100652582	rs10512255	A	G	0.443	0.058	3.311E-14	LD with rs965513
9	100652755	rs12004762	G	A	0.443	0.058	3.311E-14	LD with rs965513
9	100653128	rs12550872	A	G	0.443	0.058	3.311E-14	LD with rs965513
9	100653821	rs7034249	T	C	0.443	0.058	3.311E-14	LD with rs965513
9	100654093	rs7034648	A	C	0.443	0.058	3.311E-14	LD with rs965513
9	100656459	rs7032114	G	T	0.443	0.058	3.311E-14	LD with rs965513
9	100657119	rs10115216	G	T	0.443	0.058	3.311E-14	LD with rs965513
9	100657720	rs7036589	T	A	0.443	0.058	3.311E-14	LD with rs965513
9	100648631	rs10818213	C	T	0.444	0.059	3.432E-14	LD with rs965513
9	100658318	rs7037324	G	A	0.442	0.058	3.643E-14	LD with rs965513
9	100556972	rs10759944	G	A	0.443	0.059	3.867E-14	LD with rs965513
9	100629827	rs4582663	G	T	0.438	0.059	8.244E-14	LD with rs965513
9	100614140	rs3758249	C	T	0.433	0.058	9.782E-14	LD with rs965513
9	100617479	rs1443434	T	G	0.433	0.058	9.796E-14	LD with rs965513
9	100610652	rs7851552	A	C	0.433	0.058	1.014E-13	LD with rs965513
9	100610759	rs7851660	A	C	0.433	0.058	1.014E-13	LD with rs965513
9	100611233	rs12348304	C	T	0.433	0.058	1.014E-13	LD with rs965513
9	100612270	rs894673	T	A	0.433	0.058	1.014E-13	LD with rs965513
9	100613700	rs3758251	C	G	0.433	0.058	1.014E-13	LD with rs965513
9	100613956	rs10046805	A	G	0.433	0.058	1.014E-13	LD with rs965513
9	100615553	rs13302470	G	A	0.432	0.058	1.035E-13	LD with rs965513
9	100615914	rs1867277	G	A	0.432	0.058	1.035E-13	LD with rs965513

© 2022 Feng X et al. *JAMA Network Open.*

9	100616066	rs1867280	C	G	0.432	0.058	1.035E-13	LD with rs965513
9	100615117	rs907577	T	C	0.432	0.058	1.057E-13	LD with rs965513
9	100615949	rs1867278	A	C	0.432	0.058	1.057E-13	LD with rs965513
9	100641771	rs756396839	C	CCACCA	0.437	0.059	1.071E-13	LD with rs965513
9	100610348	rs10818133	G	C	0.432	0.058	1.079E-13	LD with rs965513
9	100610382	rs12341377	G	C	0.432	0.058	1.079E-13	LD with rs965513
9	100588839	rs7848973	G	A	0.437	0.059	1.086E-13	LD with rs965513
9	100583074	rs10124220	C	T	0.437	0.059	1.099E-13	LD with rs965513
9	100614601	rs3758248	A	G	0.432	0.058	1.102E-13	LD with rs965513
9	100617021	rs3021526	T	C	0.432	0.058	1.119E-13	LD with rs965513
9	100608682	rs12348691	A	G	0.432	0.058	1.122E-13	LD with rs965513
9	100608980	rs13288000	C	T	0.432	0.058	1.122E-13	LD with rs965513
9	100609230	rs7873389	T	C	0.432	0.058	1.198E-13	LD with rs965513
9	100645728	rs2120264	G	A	0.435	0.059	1.217E-13	LD with rs965513
9	100634150	rs752847755	C	CAG	0.435	0.059	1.233E-13	LD with rs965513
9	100660136	rs35324451	T	A	0.433	0.058	1.268E-13	LD with rs965513
9	100632606	rs7868534	A	G	0.434	0.059	1.282E-13	LD with rs965513
9	100591705	rs7033765	T	A	0.436	0.059	1.295E-13	LD with rs965513
9	100637859	rs10123699	G	A	0.433	0.059	1.347E-13	LD with rs965513
9	100638420	rs4743139	G	A	0.435	0.059	1.348E-13	LD with rs965513
9	100634579	rs1955143	T	A	0.433	0.059	1.363E-13	LD with rs965513
9	100635780	rs10759981	G	A	0.433	0.059	1.363E-13	LD with rs965513

© 2022 Feng X et al. *JAMA Network Open.*

9	100636398	rs925487	T	C	0.433	0.059	1.363E-13	LD with rs965513
9	100647545	rs4255258	G	A	0.434	0.059	1.373E-13	LD with rs965513
9	100624259	rs12343182	C	G	0.433	0.059	1.401E-13	LD with rs965513
9	100626884	rs7860144	G	A	0.433	0.059	1.471E-13	LD with rs965513
9	100627562	rs13299924	T	C	0.433	0.059	1.471E-13	LD with rs965513
9	100628238	rs10120412	C	T	0.433	0.059	1.471E-13	LD with rs965513
9	100628268	rs10122541	A	G	0.433	0.059	1.471E-13	LD with rs965513
9	100628642	rs2417575	A	G	0.433	0.059	1.471E-13	LD with rs965513
9	100628707	rs2417576	T	C	0.433	0.059	1.471E-13	LD with rs965513
9	100631298	rs1443436	A	T	0.433	0.059	1.471E-13	LD with rs965513
9	100631662	rs7037175	C	G	0.433	0.059	1.471E-13	LD with rs965513
9	100631799	9:100631799_CCAAA_C	C	CCAAA	0.433	0.059	1.471E-13	LD with rs965513
9	100633386	rs10818175	C	T	0.433	0.059	1.471E-13	LD with rs965513
9	100634589	rs1955144	G	A	0.433	0.059	1.471E-13	LD with rs965513
9	100634751	rs1955145	A	C	0.433	0.059	1.471E-13	LD with rs965513
9	100607497	rs6478437	G	A	0.430	0.058	1.578E-13	LD with rs965513
9	100607837	rs2401639	C	T	0.430	0.058	1.578E-13	LD with rs965513
9	100639065	rs12342417	A	G	0.432	0.059	1.619E-13	LD with rs965513
9	100639275	rs10984103	C	A	0.432	0.059	1.619E-13	LD with rs965513
9	100644939	rs4743142	A	G	0.432	0.059	1.629E-13	LD with rs965513
9	100604213	rs7040492	G	A	0.429	0.058	1.737E-13	LD with rs965513

© 2022 Feng X et al. *JAMA Network Open.*

9	100585506	rs4297160	A	G	0.433	0.059	1.786E-13	LD with rs965513
9	100623377	rs993501	A	G	0.431	0.059	1.839E-13	LD with rs965513
9	100624299	rs6478445	G	A	0.431	0.059	1.839E-13	LD with rs965513
9	100624622	rs10113884	T	C	0.431	0.059	1.839E-13	LD with rs965513
9	100642065	rs7044121	T	A	0.431	0.059	1.844E-13	LD with rs965513
9	100624770	rs10117642	C	T	0.431	0.059	1.931E-13	LD with rs965513
9	100624981	rs10119853	A	G	0.431	0.059	1.931E-13	LD with rs965513
9	100625193	rs10759975	C	T	0.431	0.059	1.931E-13	LD with rs965513
9	100630439	rs7847010	C	T	0.431	0.059	1.931E-13	LD with rs965513
9	100637681	rs13287360	G	A	0.431	0.059	1.931E-13	LD with rs965513
9	100640605	rs13286291	C	A	0.431	0.059	1.937E-13	LD with rs965513
9	100624602	rs10119760	C	G	0.430	0.059	1.985E-13	LD with rs965513
9	100640410	rs4592135	A	G	0.435	0.059	2.006E-13	LD with rs965513
9	100591463	rs7045138	T	C	0.432	0.059	2.040E-13	LD with rs965513
9	100599969	rs7049054	G	T	0.428	0.058	2.144E-13	LD with rs965513
9	100600241	rs7018683	C	T	0.428	0.058	2.144E-13	LD with rs965513
9	100600702	rs7860598	A	G	0.428	0.058	2.144E-13	LD with rs965513
9	100598568	rs4743134	A	C	0.427	0.058	2.200E-13	LD with rs965513
9	100599493	rs4743135	T	C	0.427	0.058	2.200E-13	LD with rs965513
9	100600635	rs7847126	C	A	0.427	0.058	2.200E-13	LD with rs965513
9	100610859	rs10638868	ACTCC	A	0.428	0.058	2.360E-13	LD with rs965513
9	100596439	rs7043885	T	C	0.427	0.058	2.410E-13	LD with rs965513

9	100603008	rs10818124	T	C	0.426	0.058	2.412E-13	LD with rs965513
9	100619090	rs202204950	T	TCTC	0.427	0.058	2.850E-13	LD with rs965513
9	100622189	rs907581	G	A	0.427	0.058	2.892E-13	LD with rs965513
9	100617583	rs1443435	C	T	0.424	0.058	3.067E-13	LD with rs965513
9	100596689	9:100596689_TA_T	T	TA	0.446	0.061	3.394E-13	LD with rs965513
9	100601771	rs7026669	C	T	0.439	0.060	3.736E-13	LD with rs965513
9	100606559	9:100606559_AAAAT_A	A	AAAAT	0.437	0.060	5.064E-13	LD with rs965513
9	100606216	rs13295389	A	G	0.437	0.060	5.171E-13	LD with rs965513
9	100620077	rs35828132	ACCGG	A	0.420	0.058	6.287E-13	LD with rs965513
9	100582390	rs35061703	TAGAC	T	0.438	0.061	6.698E-13	LD with rs965513
9	100638602	rs577883824	G	GT	0.424	0.059	6.956E-13	LD with rs965513
9	100612185	rs894672	T	C	0.432	0.060	8.192E-13	LD with rs965513
9	100612774	rs7848950	C	T	0.432	0.060	8.192E-13	LD with rs965513
9	100614188	rs10983975	C	A	0.432	0.060	8.192E-13	LD with rs965513
9	100614296	rs4743138	G	A	0.432	0.060	8.192E-13	LD with rs965513
9	100615359	rs907576	T	C	0.432	0.060	8.192E-13	LD with rs965513
9	100615660	rs7849497	C	G	0.432	0.060	8.192E-13	LD with rs965513
9	100616041	rs1867279	C	T	0.432	0.060	8.192E-13	LD with rs965513
9	100616583	rs3021523	C	T	0.432	0.060	8.317E-13	LD with rs965513
9	100612807	rs1348386	G	A	0.431	0.060	8.724E-13	LD with rs965513
9	100617375	rs7046645	C	T	0.432	0.060	8.739E-13	LD with rs965513

© 2022 Feng X et al. *JAMA Network Open.*

9	100619719	rs12347191	T	C	0.417	0.058	8.775E-13	LD with rs965513
9	100620412	rs4460498	C	T	0.417	0.058	9.043E-13	LD with rs965513
9	100606188	rs7849834	C	T	0.431	0.060	1.049E-12	LD with rs965513
9	100606036	rs7875783	G	A	0.429	0.060	1.222E-12	LD with rs965513
9	100605433	rs7048394	C	T	0.429	0.060	1.229E-12	LD with rs965513
9	100581427	rs7870871	C	T	0.429	0.060	1.270E-12	LD with rs965513
9	100606004	rs7860651	T	C	0.428	0.060	1.388E-12	LD with rs965513
9	100603935	rs7023267	A	G	0.428	0.060	1.465E-12	LD with rs965513
9	100600308	rs7046186	G	A	0.427	0.061	1.748E-12	LD with rs965513
9	100600712	rs7847259	G	A	0.427	0.061	1.748E-12	LD with rs965513
9	100595238	rs7024345	G	A	0.427	0.061	1.760E-12	LD with rs965513
9	100644845	9:100644845_TA_T	T	TA	0.429	0.061	1.778E-12	LD with rs965513
9	100592705	rs10739513	T	C	0.427	0.061	1.930E-12	LD with rs965513
9	100598261	rs1912996	T	G	0.426	0.061	2.000E-12	LD with rs965513
9	100658123	rs7021160	A	C	0.425	0.061	2.129E-12	LD with rs965513
9	100592030	rs6478423	T	C	0.426	0.061	2.187E-12	LD with rs965513
9	100620730	rs973473	G	T	0.424	0.061	2.897E-12	LD with rs965513
9	100621386	rs1912998	C	T	0.423	0.061	3.508E-12	LD with rs965513
9	100598842	9:100598842_CT_C	C	CT	0.413	0.059	3.756E-12	LD with rs965513
9	100622597	rs907580	C	T	0.421	0.061	4.069E-12	LD with rs965513
9	100622723	rs907579	T	G	0.421	0.061	4.069E-12	LD with rs965513
9	100622883	rs907578	G	A	0.421	0.061	4.069E-12	LD with rs965513

© 2022 Feng X et al. *JAMA Network Open.*

9	100620326	rs1465965	T	C	0.421	0.061	4.335E-12	LD with rs965513
9	100621926	rs907582	C	T	0.420	0.061	4.772E-12	LD with rs965513
9	100608745	rs10759960	G	A	0.409	0.059	5.052E-12	LD with rs965513
9	100619733	rs925486	T	C	0.418	0.061	5.681E-12	LD with rs965513
9	100619561	rs925485	C	G	0.418	0.061	6.015E-12	LD with rs965513
9	100669073	rs723227	A	G	0.401	0.059	8.071E-12	LD with rs965513
9	100534823	rs7045465	T	A	0.391	0.058	1.768E-11	LD with rs965513
9	100534147	rs1877431	G	A	0.389	0.058	2.337E-11	LD with rs965513
9	100575888	rs7859751	A	G	0.398	0.060	2.364E-11	LD with rs965513
9	100574120	rs7870795	C	T	0.398	0.060	2.406E-11	LD with rs965513
9	100542706	rs6478395	C	T	-0.406	0.061	2.495E-11	LD with rs965513
9	100573925	rs7870540	C	T	0.397	0.060	3.016E-11	LD with rs965513
9	100532965	rs1533180	G	T	-0.396	0.061	1.118E-10	LD with rs965513
9	100533317	9:100533317_ACT_A	A	ACT	0.371	0.059	2.362E-10	LD with rs965513
9	100551908	rs7847449	C	A	-0.380	0.064	3.485E-09	LD with rs965513
9	100561147	rs1867281	A	G	-0.374	0.063	3.733E-09	LD with rs965513
9	100560368	rs10983796	T	C	-0.372	0.063	4.278E-09	LD with rs965513
9	100536542	rs62573974	C	T	-0.363	0.063	7.001E-09	LD with rs965513
9	100539696	rs10983705	G	C	-0.362	0.063	7.403E-09	LD with rs965513
9	100546486	rs754459513	TAA	T	-0.360	0.063	8.816E-09	LD with rs965513
9	100675976	rs2120262	A	C	0.337	0.059	8.829E-09	LD with rs965513

© 2022 Feng X et al. *JAMA Network Open.*

9	100543880	rs1877432	G	A	-0.360	0.063	9.588E-09	LD with rs965513
9	100536605	9:100536605_TGAAAAGTA_T	TGAAAAGTA	T	-0.359	0.063	9.629E-09	LD with rs965513
9	100671393	rs10760011	C	A	0.332	0.058	1.326E-08	LD with rs965513
2	218296732	rs16857611	C	T	0.349	0.062	2.093E-08	LD with rs6759952
2	218296508	rs16857609	C	T	0.349	0.062	2.148E-08	LD with rs6759952
2	218296374	rs57481445	A	G	0.347	0.062	2.418E-08	LD with rs6759952
9	100670610	rs7042459	C	T	0.321	0.059	4.091E-08	LD with rs965513
2	218294217	rs12990503	G	C	0.341	0.062	4.252E-08	LD with rs6759952
2	218292141	rs3821098	C	T	0.340	0.062	4.521E-08	LD with rs6759952
2	218292158	rs11693806	G	C	0.340	0.062	4.521E-08	LD with rs6759952

CHR=chromosome; SNP=single nucleotide polymorphisms; MAF=minor allele frequency.

Note: In the GWAS of UK Biobank, we identified 223 genetic loci associated with TC risk of P < 5 × 10-8 on chromosome 2 and 9, which were in extremely strong LD with rs965513 on chromosome 9 and rs6759952 on chromosome 2 (known SNP related with TC, r2=0.99). The GWAS result was presented in Supplementary Table S3, with the threshold $P < 5 \times 10^{-8}$. For replication, we conducted a GWAS meta-analysis using GWAS summary statistics from UK Biobank, the FinnGen Study and Italian residents.

© 2022 Feng X et al. *JAMA Network Open.*

eTable 6 Meta-GWAS results of three cohorts (n=1956)

CHR	Position	SNP	Effect_allele	Other_allele	BETA	SE	*P*	Q	R^2	N	I	MAF	Gene[b]	LD
18	648465	rs11877057	G	A	0.261	0.062	2.84E-05	0.3641	0.12%	3	1.03	0.06	*C18orf56, CLUL1*	
9	100726574	rs4743149	A	G	0.262	0.058	5.32E-06	0.7087	0.12%	3	0	0.06	*ANP32B, HEMGN*	LD with rs10512256
6	139151784	rs11155012	A	G	0.173	0.042	3.89E-05	0.8862	0.18%	3	0	0.28	*ECT2L*	
9	109689859	rs3814540	C	T	0.161	0.039	4.53E-05	0.483	0.19%	3	0	0.39	*MIR548Q, ZNF462*	
6	139143088	rs7773177	G	A	0.175	0.042	3.28E-05	0.8323	0.19%	3	0	0.28	*ECT2L*	LD with rs11155012
10	79595473	rs1781797	A	G	-0.177	0.043	3.96E-05	0.6903	0.19%	3	0	0.27	*DLG5*	
14	36633415	rs1169148	T	C	0.178	0.043	4.26E-05	0.9167	0.19%	3	0	0.27	*LINC00609, PTCSC3*	LD with rs1951375
14	36558089	rs8007617	G	A	0.164	0.040	3.68E-05	0.6218	0.19%	3	0	0.39	*LINC00609*	LD with rs1951375
19	9264681	rs8112516	G	A	0.200	0.046	1.59E-05	0.5166	0.19%	3	0	0.2	*ZNF317*	
7	152178955	rs10235893	G	A	0.173	0.042	3.67E-05	0.9873	0.19%	3	0	0.31	*LINC01003*	
10	27734691	rs17681969	C	T	-0.197	0.046	1.72E-05	0.3469	0.20%	3	5.55	0.22	*PTCHD3*	LD with rs2429499
11	62212013	rs7942143	T	G	-0.211	0.051	4.02E-05	0.7887	0.21%	3	0	0.19	*AHNAK*	

© 2022 Feng X et al. *JAMA Network Open.*

14	36520775	rs1951375	T	C	-0.169	0.039	1.54E-05	0.3941	0.21%	3	0	0.42	*LOC105370452*	
10	27700887	rs2429499	A	G	0.187	0.043	1.10E-05	0.4367	0.21%	3	0	0.28	*PTCHD3*	
14	36579930	rs7145145	G	A	0.172	0.040	1.44E-05	0.6312	0.21%	3	0	0.4	*LINC00609*	LD with rs1951375
10	27704360	rs7915825	T	G	-0.212	0.048	1.04E-05	0.4755	0.22%	3	0	0.2	*PTCHD3*	LD with rs2429499
8	32317917	rs17716295	A	C	0.185	0.040	3.84E-06	0.9246	0.23%	3	0	0.33	*NRG1, NRG1-IT3*	
14	36564242	rs116228885	C	T	0.178	0.039	4.77E-06	0.6424	0.24%	3	0	0.43	*LINC00609*	LD with rs1951375
5	85389354	rs9293456	G	A	0.310	0.071	1.13E-05	0.6453	0.24%	3	0	0.09	*intergenic variant*	
2	218292652	rs1478581	A	G	0.236	0.047	6.95E-07	0.7348	0.25%	3	0	0.18	*DIRC3*	LD with rs6759952
18	674320	rs3744962	G	A	0.320	0.062	2.72E-07	0.8305	0.25%	3	0	0.09	*ENOSF1, TYMS*	LD with rs11877057
2	218310340	rs966423	T	C	-0.187	0.038	1.05E-06	0.8997	0.26%	3	0	0.43	*DIRC3*	LD with rs6759952
14	36583905	rs7150768	T	G	0.193	0.040	1.17E-06	0.7552	0.27%	3	0	0.4	*LINC00609*	LD with rs1951375
9	100686815	rs10512256	G	T	0.269	0.057	2.73E-06	0.6822	0.28%	3	0	0.15	*TRMO*	
5	111463837	rs27982	C	T	-0.281	0.054	2.18E-07	0.6557	0.30%	3	0	0.15	*EPB41L4A*	

© 2022 Feng X et al. *JAMA Network Open.*

5	111495150	rs7730334	G	A	-0.298	0.058	2.78E-07	0.8121	0.30%	3	0	0.13	*EPB41L4A-AS1*	LD with rs27982
14	36640601	rs1169151	A	G	0.205	0.039	1.90E-07	0.9057	0.31%	3	0	0.42	*PTCSC3*	LD with rs1951375
9	100742253	rs7855088	T	C	-0.209	0.038	4.91E-08	0.4237	0.32%	3	0	0.41	*ANP32B*	LD with rs965513
2	218277119	rs12478389	T	C	0.353	0.061	6.72E-09	0.7809	0.34%	3	0	0.1	*DIRC3*	LD with rs6759952
9	100664194	rs7040232	G	A	-0.259	0.047	3.69E-08	0.4067	0.38%	3	0	0.25	*C9orf156*	LD with rs965513
2	218305064	rs6715218	C	T	0.252	0.040	3.76E-10	0.99	0.40%	3	0	0.3	*DIRC3*	LD with rs6759952
5	111480534	rs17134154	C	T	-0.319	0.059	5.81E-08	0.7168	0.43%	3	0	0.17	*EPB41L4A-AS1*	LD with rs27982
2	218271719	rs6759952	C	T	-0.253	0.038	4.36E-11	0.3397	0.48%	3	7.37	0.44	*DIRC3*	
2	218304106	rs2618148	T	C	0.278	0.040	5.04E-12	0.8924	0.50%	3	0	0.31	*DIRC3*	LD with rs6759952
9	100666931	rs6586	T	C	-0.304	0.040	2.45E-14	0.9839	0.66%	3	0	0.38	*C9orf156*	LD with rs965513
9	100608682	rs12348691	A	G	-0.450	0.039	2.61E-31	0.8897	1.48%	3	0	0.42	*FOXE1*	LD with rs965513
9	100636398	rs925487	T	C	-0.472	0.039	7.52E-34	0.7563	1.55%	3	0	0.37	*FOXE1*	LD with rs965513

© 2022 Feng X et al. *JAMA Network Open.*

9	100650096	rs7866436	A	G	-0.473	0.039	3.30E-34	0.75	1.56%	3	0	0.37	*C9orf156*	LD with rs965513
9	100667599	rs1561961	T	C	-0.476	0.039	1.63E-34	0.7056	1.58%	3	0	0.37	*C9orf156*	LD with rs965513
9	100588839	rs7848973	G	A	-0.491	0.039	5.85E-37	0.6598	1.74%	3	0	0.41	*FOXE1*	LD with rs965513
9	100556109	rs965513	G	A	-0.519	0.039	5.60E-40	0.5895	1.80%	3	0	0.34	*FOXE1*	

CHR=chromosome; SNP=single nucleotide polymorphisms; MAF=minor allele frequency.

Note: This meta-analysis was conducted using a fixed-effect inverse variance weighted method, and significant SNPs in three cohorts with $P < 5 \times 10^{-5}$ and Q (the Cochran's Q-test) > 0.05 were considered.

a Quality control as following: MAF < 0.01, LD >= 0.01, Q (the Cochran's Q-test) > 0.05, N=3

b No relevant genes were found in the set upstream and downstream range of 100kb

© 2022 Feng X et al. *JAMA Network Open.*

eTable 7 Genetic risk score selection					
PRS[a]	SNP	Effect allele	Beta[a]	*P*	Origin
PRS1[b]	rs6759952	C	-0.253	4.36E-11	Meta-GWAS, P < 5E-5
	rs9293456	G	0.310	1.13E-05	Meta-GWAS, P < 5E-5
	rs27982	C	-0.281	2.18E-07	Meta-GWAS, P < 5E-5
	rs7773177	G	0.175	3.28E-05	Meta-GWAS, P < 5E-5
	rs10235893	G	0.173	3.67E-05	Meta-GWAS, P < 5E-5
	rs17716295	A	0.185	3.84E-06	Meta-GWAS, P < 5E-5
	rs965513	G	-0.519	5.60E-40	Meta-GWAS, P < 5E-5
	rs10512256	G	0.269	2.73E-06	Meta-GWAS, P < 5E-5
	rs3814540	C	0.161	4.53E-05	Meta-GWAS, P < 5E-5
	rs2429499	A	0.187	1.10E-05	Meta-GWAS, P < 5E-5
	rs1781797	A	-0.177	3.96E-05	Meta-GWAS, P < 5E-5
	rs7942143	T	-0.211	4.02E-05	Meta-GWAS, P < 5E-5
	rs1951375	T	-0.169	1.54E-05	Meta-GWAS, P < 5E-5
	rs11877057	G	0.261	2.84E-05	Meta-GWAS, P < 5E-5
	rs8112516	G	0.200	1.59E-05	Meta-GWAS, P < 5E-5
PRS2[b]	rs6759952	C	-0.253	4.36E-11	Meta-GWAS, P < 5E-6
	rs27982	C	-0.281	2.18E-07	Meta-GWAS, P < 5E-6
	rs17716295	A	0.185	3.84E-06	Meta-GWAS, P < 5E-6
	rs965513	G	-0.519	5.60E-40	Meta-GWAS, P < 5E-6
	rs10512256	G	0.269	2.73E-06	Meta-GWAS, P < 5E-6
	rs11622885	C	0.178	4.77E-06	Meta-GWAS, P < 5E-6
	rs3744962	G	0.320	2.72E-07	Meta-GWAS, P < 5E-6
PRS3[b]	rs10069690	T	0.182	3.20E-07	28195142
	rs10238549	C	0.239	4.10E-06	23894154
	rs10415826	T	0.199	8.70E-07	33527407
	rs10781500	C	0.207	3.50E-05	23894154
	rs116909374	T	0.593	5.00E-11	23894154
	rs12129938	A	0.278	4.00E-11	28195142
	rs16950982	G	0.199	4.70E-09	33527407
	rs2439302	G	0.307	2.00E-09	22267200
	rs334729	G	-0.635	8.70E-08	33527407
	rs368187	G	0.329	5.00E-23	28195142
	rs555678255	C	0.672	8.00E-07	32887889
	rs56062135	T	0.215	5.00E-09	28195142
	rs6759952	T	0.223	6.00E-10	23894154
	rs6793295	T	0.207	3.00E-08	28195142
	rs7030280	C	0.568	5.00E-28	32887889
	rs7267944	C	0.278	1.34E-08	25029422
	rs72806259	G	-0.398	5.00E-07	32887889
	rs73227498	A	0.315	3.00E-10	28195142

© 2022 Feng X et al. *JAMA Network Open.*

	rs74518511	C	-0.854	9.00E-07	32887889
	rs76032629	G	-0.519	6.00E-07	32887889
	rs7617304	T	0.223	4.60E-05	23894154
	rs77166399	G	-0.331	9.00E-07	32887889
	rs7902587	T	0.344	5.00E-11	28195142
PRS4[b]	rs368187	G	0.329	5.10E-23	32132206
	rs12129938	A	0.278	4.00E-11	32132206
	rs11693806	C	0.358	1.50E-24	32132206
	rs6793295	T	0.207	2.70E-08	32132206
	rs73227498	A	0.315	3.00E-10	32132206
	rs2466076	G	0.278	1.50E-17	32132206
	rs1588635	A	0.525	2.00E-58	32132206
	rs2289261	C	0.207	3.10E-09	32132206
	rs7902587	T	0.344	5.40E-11	32132206
	rs116909374	T	0.593	1.10E-16	32132206

SNP=single nucleotide polymorphisms; PRS=polygenic risk score

a The SNPs constructing PRS were performed quality control as following: MAF<0.01 and LD=0.01, and beta coefficient for effect size used as weight in PRS

b PRS1 (P < 5E-6) and PRS2 (P < 5E-5) were derived from our meta-GWAS analysis; PRS3 was used SNPs from Table S2 and excluded LD (LD=0.01); and PRS4 is used for next analysis

© 2022 Feng X et al. *JAMA Network Open.*

eTable 8 Association of different PRSs and risk of thyroid cancer

Characteristics	Model 1[a]					Model 2[a]			
	HR (95%CI)	*P*	*P* for trend	HR (95%CI)[b]		HR (95%CI)	*P*	*P* for trend	HR (95%CI)[b]
Weighted PRS2 (7 SNPs)			2.61 E-14	2.05 (1.46, 2.9)				1.37 E-13	2.33 (1.93, 2.82)
T1	Reference					Reference			
T2	1.84 (0.57, 5.94)	0.31				1.31 (0.99, 1.73)	0.06		
T3	2.55 (0.81, 7.99)	0.11				2.41 (1.88, 3.08)	3.36 E-12		
Unweighted PRS2 (7 SNPs)			0.08	1.01 (0.93, 1.1)				0.15	1.05 (0.99, 1.12)
T1	Reference					Reference			
T2	1.03 (0.75, 1.43)	0.84				1.04 (0.83, 1.29)	0.76		
T3	1.03 (0.72, 1.49)	0.86				1.22 (0.93, 1.6)	0.15		
Weighted PRS3 (23 SNPs)			1.36 E-12	1.66 (1.42, 1.96)				1.93 E-11	1.95 (1.68, 2.26)
T1	Reference					Reference			
T2	1.81 (1.02, 3.2)	0.04				1.47 (1.11, 1.95)	0.01		
T3	2.8 (1.65, 4.77)	1.44 E-04				2.68 (2.07, 3.47)	6.69 E-14		
Unweighted PRS3 (23 SNPs)			7.45 E-08	1.03 (0.98, 1.08)				1.17 E-07	1.05 (1.01, 1.09)
T1	Reference					Reference			

© 2022 Feng X et al. *JAMA Network Open.*

T2	0.96 (0.65, 1.42)	0.85				1.1 (0.86, 1.4)	0.45		
T3	1.07 (0.75, 1.52)	0.73				1.31 (1.03, 1.66)	0.03		
Weighted PRS4 (10 SNPs)			4.35 E-17	1.77 (1.4, 2.24)				1.74 E-15	1.74 (1.56, 1.94)
T1	Reference					Reference			
T2	1.04 (0.59, 1.84)	0.89				1.4 (1.06, 1.84)	0.02		
T3	1.72 (1.03, 2.88)	0.04				2.28 (1.77, 2.94)	1.32 E-10		
Unweighted PRS4 (10 SNPs)			0.01	1.15 (1.06, 1.24)				0.03	1.18 (1.12, 1.24)
T1	Reference					Reference			
T2	1.75 (1.07, 2.87)	0.03				1.28 (0.99, 1.66)	0.06		
T3	1.88 (1.17, 3.02)	0.01				1.88 (1.47, 2.4)	4.42 E-07		

SNP=single nucleotide polymorphisms; PRS=polygenic risk score.

[a] Model 1 was not adjustment, Model 2 was adjusted for age, sex, and genetic composition, townsend deprivation index at recruitment, qualifications and average total household income before tax

[b] PRS were performed as a continuous variable using logistic regression model

© 2022 Feng X et al. *JAMA Network Open.*

eTable 9 Baseline characteristics of thyroid cancer participants between FTC and PTC

Variable	FTC (n = 55)	PTC (n = 207)	*P*
Age	57.44 (7.75)	56.59 (7.89)	0.48
Sex			0.75
Women	38 (69.09)	150 (72.46)	
Men	17 (30.91)	57 (27.54)	
Townsend deprivation index			0.67
1 (least deprived)	9 (16.36)	38 (18.36)	
2-4	37 (67.27)	126 (60.87)	
5 (most deprived)	9 (16.36)	43 (20.77)	
Educational qualifications			0.06
College or University degree	19 (34.55)	61 (29.47)	
Secondary Education	26 (47.27)	128 (61.84)	
Some professional qualifications	10 (18.18)	18 (8.70)	
Average total household income before tax			0.80
Less than 18,000	9 (16.36)	38 (18.36)	
18,000 to 30,999	15 (27.27)	52 (25.12)	
31,000 to 51,999	21 (38.18)	87 (42.03)	
52,000 to 100,000	9 (16.36)	23 (11.11)	
Greater than 100,000	1 (1.82)	7 (3.38)	
Diet index			0.63
Unfavourable	29 (52.73)	119 (57.49)	
Favourable	26 (47.27)	88 (42.51)	
Total moderate-vigorous physical activity			0.81
Favourable	25 (45.45)	88 (42.51)	
Unfavourable	30 (54.55)	119 (57.49)	
Smoke intake			0.35
Favourable	51 (92.73)	180 (86.96)	
Unfavourable	4 (7.27)	27 (13.04)	
Alcohol consumption			0.18
Favourable	47 (85.45)	157 (75.85)	
Unfavourable	8 (14.55)	50 (24.15)	
Healthy weight			0.23
Unfavourable	24 (43.64)	70 (33.82)	
Favourable	31 (56.36)	137 (66.18)	
Lifestyle			0.26
Favourable	19 (34.55)	62 (29.95)	
Intermediate	23 (41.82)	72 (34.78)	
Unfavourable	13 (23.64)	73 (35.27)	
Weighted lifestyle			0.76
Favourable	13 (23.64)	47 (22.71)	

© 2022 Feng X et al. *JAMA Network Open.*

Intermediate	21 (38.18)	70 (33.82)		
Unfavourable	21 (38.18)	90 (43.48)		
Weighted PRS1			0.82	
T1	9 (16.36)	29 (14.01)		
T2	19 (34.55)	67 (32.37)		
T3	27 (49.09)	111 (53.62)		

© 2022 Feng X et al. *JAMA Network Open.*

eTable 10 The additive interaction between lifestyle and PRS (RERI)[a]

Characteristics	PRS T2		PRS T3	
	RERI	95%CI	RERI	95%CI
Weighted healthy lifestyle				
Favourable	Reference		Reference	
Intermediate	1.16	0.20,2.12	0.37	-0.81,1.54
Unfavourable	1.01	-0.03,2.06	1.90	0.44,3.35
Unweighted healthy lifestyle				
Favourable	Reference		Reference	
Intermediate	0.96	0.17,1.75	0.33	-0.67,1.33)
Unfavourable	-0.02	-1.02,0.98	0.72	-0.48,1.93)
Diet index				
Favourable	Reference		Reference	
Unfavourable	-0.46	-1.43,0.50	-0.76	-1.95,0.44
Total moderate-vigorous physical activity				
Favourable	Reference		Reference	
Unfavourable	0.48	-0.34,1.30	0.66	-0.32,1.63
Smoke intake				
Favourable	Reference		Reference	
Unfavourable	0.46	-1.11,2.03	2.79	0.45,5.12
Alcohol consumption				
Favourable	Reference		Reference	
Unfavourable	-0.77	-1.60,0.06	-0.59	-1.53,0.36
Healthy weight				
Favourable	Reference		Reference	
Unfavourable	0.51	-0.22,1.23	0.62	-0.28,1.52

PRS=polygenic risk score; RERI=relative excess risk.

[a]adjusted for age, sex, and genetic composition, townsend deprivation index at recruitment, qualifications and average total household income before tax.

© 2022 Feng X et al. *JAMA Network Open.*

eTable 11 Associations of lifestyle components with incident thyroid cancer according to PRS stratified analysis in the nested case-control design[a]

Characteristics	PRS T1			PRS T2			PRS T3		*P* for interaction
	OR (95%CI)	*P*		OR (95%CI)	*P*		OR (95%CI)	*P*	
Weighted lifestyle									0.14
Favourable	Reference			Reference			Reference		
Intermediate	0.95 (0.33, 2.74)	0.92		2.50 (1.14, 5.47)	0.02		1.03 (0.63, 1.69)	0.90	
$RERI_{intermediate/favorable}$	Reference			1.16 (0.35, 1.97)			0.02 (-1.37, 1.41)		
Unfavourable	1.48 (0.46, 4.73)	0.51		2.67 (1.15, 6.20)	0.023		2.05 (1.21, 3.47)	0.01	
RERI Unfavourable/Favourable	Reference			0.15 (-0.69, 0.99)			1.08 (-0.12, 2.28)		
Unweighted lifestyle									0.28
Favourable	Reference			Reference			Reference		
Intermediate	0.23 (0.07, 0.72)	0.012		2.21 (1.07, 4.54)	0.031		1.15 (0.70, 1.88)	0.58	
RERI intermediate/Favourable	Reference			1.63 (0.61, 2.65)			0.71 (-0.17, 1.59)		
Unfavourable	0.94 (0.35, 2.54)	0.90		2.38 (1.11, 5.08)	0.03		1.97 (1.18, 3.30)	0.01	
RERI Unfavourable/Favourable	Reference			0.15 (-0.69, 0.99)			1.08 (-0.12, 2.28)		
Diet index									0.45
Favourable	Reference			Reference			Reference		
Unfavourable	1.40 (0.63, 3.11)	0.41		1.48 (0.84, 2.62)	0.18		0.99 (0.66, 1.49)	0.96	
RERI[£]				-0.56 (-1.66, 0.54)			-0.40 (-1.50, 0.70)		

© 2022 Feng X et al. *JAMA Network Open.*

Total moderate-vigorous physical activity									0.52
Favourable	Reference			Reference			Reference		
Unfavourable	0.90 (0.41, 1.99)	0.80		2.14 (1.20, 3.82)	0.010		1.51 (1.01, 2.25)	0.04	
RERI				0.95 (0.25, 1.65)			0.93 (-0.06, 1.91)		
Smoke intake									0.06
Favourable	Reference			Reference			Reference		
Unfavourable	0.65 (0.11, 3.94)	0.64		0.80 (0.29, 2.16)	0.657		2.43 (1.19, 4.97)	0.02	
RERI				-0.213 (-1.880, 1.454)			3.406 (0.547, 6.264)		
Alcohol consumption									0.60
Favourable	Reference			Reference			Reference		
Unfavourable	0.88 (0.38, 2.04)	0.76		0.73 (0.36, 1.48)	0.38		0.88 (0.55, 1.42)	0.60	
RERI				-0.547 (-1.353, 0.258)			-0.05 (-0.96, 0.85)		
Weight									0.36
Favourable	Reference			Reference			Reference		
Unfavourable	0.96 (0.42, 2.20)	0.92		1.72 (0.94, 3.15)	0.08		1.44 (0.94, 2.21)	0.10	
RERI				0.77 (0.14, 1.40)			0.66 (-0.31, 1.64)		
PRS=polygenic risk score; RERI=relative excess risk.									
[a]adjusted for age, sex, and genetic composition, townsend deprivation index at recruitment, qualifications and average total household income before tax									
Note: RERI was used favourable lifestyle and lowest PRS as reference, and described as beta (95% CI)									

© 2022 Feng X et al. *JAMA Network Open.*

eTable 12 Combined analysis of PRS and lifestyle components on the risk of thyroid cancer*								
Characteristics	PRS T1			PRS T2			PRS T3	
	HR (95%CI)	*P*		HR (95%CI)	*P*		HR (95%CI)	*P*
Weighted healthy lifestyle								
Favourable	Reference			1.1 (0.61, 1.98)	0.76		2.63 (1.59, 4.35)	<0.001
Intermediate	1.35 (0.77, 2.38)	0.30		2.62 (1.58, 4.32)	<0.001		3.35 (2.06, 5.46)	<0.001
Unfavourable	1.44 (0.8, 2.6)	0.22		2.81 (1.69, 4.69)	<0.001		4.89 (3.03, 7.91)	<0.001
Unweighted healthy lifestyle								
Favourable	Reference			1.41 (0.87, 2.28)	0.16		2.64 (1.71, 4.07)	<0.001
Intermediate	0.78 (0.43, 1.41)	0.41		2.11 (1.33, 3.35)	0.00		2.72 (1.75, 4.23)	<0.001
Unfavourable	1.45 (0.86, 2.47)	0.17		1.94 (1.19, 3.17)	0.01		3.76 (2.42, 5.84)	<0.001
Diet index								
Favourable	Reference			2.02 (1.35, 3.04)	<0.001		3.56 (2.44, 5.2)	<0.001
Unfavourable	1.5 (0.95, 2.38)	0.08		2.16 (1.41, 3.3)	<0.001		3.26 (2.18, 4.86)	<0.001
Total moderate-vigorous physical activity								
Favourable	Reference			1.53 (1, 2.35)	0.05		2.82 (1.91, 4.16)	<0.001
Unfavourable	1.33 (0.84, 2.1)	0.23		2.47 (1.65, 3.7)	<0.001		3.75 (2.55, 5.49)	<0.001
Smoke intake								
Favourable	Reference			1.67 (1.24, 2.25)	<0.001		2.62 (1.98, 3.46)	<0.001
Unfavourable	0.98 (0.4, 2.41)	0.96		2.15 (1.13, 4.08)	0.02		5.54 (3.55, 8.65)	<0.001
Alcohol consumption								
Favourable	Reference			1.93 (1.39, 2.68)	<0.001		2.93 (2.15, 3.99)	<0.001
Unfavourable	0.88 (0.53, 1.48)	0.63		0.97 (0.59, 1.6)	0.91		2.24 (1.52, 3.29)	<0.001
Healthy weight								
Favourable	Reference			1.45 (0.91, 2.31)	0.12		2.6 (1.71, 3.96)	<0.001

© 2022 Feng X et al. *JAMA Network Open.*

Unfavourable	1.15 (0.72, 1.83)	0.56		2.16 (1.42, 3.28)	<0.001		3.42 (2.3, 5.09)	<0.001

PRS=polygenic risk score.

*adjusted for age, sex, genetic composition, townsend deprivation index at recruitment, qualifications and average total household income before tax.

© 2022 Feng X et al. *JAMA Network Open.*

eTable 13 The sex-difference of associations between lifestyle components with incident thyroid cancer[a]							
Characteristics	Women				Men		
	HR (95%CI)	*P*	*P* for trend[b]		HR (95%CI)	*P*	*P* for trend[b]
Total fruit and vegetables intake			0.711				0.531
Favourable	Reference				Reference		
Unfavourable	0.93 (0.73, 1.19)	0.555			0.92 (0.6, 1.42)	0.720	
Fish intake			0.989				0.976
Favourable	Reference				Reference		
Unfavourable	1.01 (0.77, 1.32)	0.959			0.86 (0.55, 1.35)	0.515	
Processed meat and red meat intake			0.707				0.259
Favourable	Reference				Reference		
Unfavourable	0.89 (0.71, 1.13)	0.353			1.46 (0.88, 2.43)	0.142	
Whole grains intake			0.221				0.393
Favourable	Reference				Reference		
Unfavourable	0.93 (0.72, 1.19)	0.572			0.95 (0.65, 1.38)	0.772	
Refine grains intake			0.552				0.884
Favourable	Reference				Reference		
Unfavourable	0.96 (0.76, 1.2)	0.700			0.9 (0.62, 1.3)	0.571	
Sugar drinking intake			0.272				0.981
Favourable	Reference				Reference		
Unfavourable	1.2 (0.87, 1.66)	0.272			1.01 (0.63, 1.59)	0.981	
Diet index			0.586				0.861
Favourable	Reference				Reference		
Unfavourable	1 (0.79, 1.26)	0.987			1.1 (0.76, 1.6)	0.598	
Total moderate-vigorous physical activity			NA				NA
Favourable	Reference				Reference		
Unfavourable	1.32 (1.05, 1.67)	0.016			1.66 (1.15, 2.39)	0.006	
Smoke intake			NA				NA
Favourable	Reference				Reference		

© 2022 Feng X et al. *JAMA Network Open.*

Unfavourable	1.36 (0.89, 2.07)	0.158			2.17 (1.33, 3.54)	0.002	
Alcohol consumption			0.025				0.214
Favourable	Reference				Reference		
Unfavourable	0.68 (0.51, 0.9)	0.006			0.76 (0.5, 1.15)	0.189	
Weight			0.003				0.045
Favourable	Reference				Reference		
Unfavourable	1.37 (1.08, 1.75)	0.010			1.17 (0.8, 1.71)	0.418	
Unweighted lifestyle			0.151				0.036
Favourable	Reference				Reference		
Intermediate	1.17 (0.89, 1.53)	0.265			0.97 (0.61, 1.55)	0.893	
Unfavourable	1.3 (0.97, 1.73)	0.076			1.56 (1.01, 2.42)	0.046	
Weighted lifestyle			<0.001				<0.001
Favourable	Reference				Reference		
Intermediate	1.63 (1.22, 2.17)	0.001			1.19 (0.68, 2.08)	0.540	
Unfavourable	1.99 (1.45, 2.71)	<0.001			1.79 (1.15, 2.8)	0.010	
PRS			<0.001				<0.001
T1	Reference				Reference		
T2	1.62 (1.15, 2.27)	0.006			1.9 (1.11, 3.27)	0.020	
T3	2.82 (2.06, 3.86)	<0.001			2.83 (1.7, 4.71)	<0.001	

PRS=polygenic risk score.

[a]P for trend were performed by using factors ascontinuous variable.

[b]adjusted for age, genetic composition, townsend deprivation index at recruitment, qualifications and average total household income before tax, and women further adjusted for age at menarche, menopausal status, number of live births, ever taken oral contraceptive pill, and ever used hormone-replacement therapy (HRT)

© 2022 Feng X et al. *JAMA Network Open.*

eTable 14 Combined analysis of PRS and lifestyle components on the risk of thyroid cancer in women[a]

Characteristics	PRS T1			PRS T2			PRS T3	
	HR (95%CI)	*P*		HR (95%CI)	*P*		HR (95%CI)	*P*
Weighted lifestyle								
Favourable	Reference			1.20 (0.59, 2.44)	0.61		2.93 (1.6, 5.36)	<0.001
Intermediate	1.61 (0.83, 3.09)	0.16		2.86 (1.57, 5.2)	<0.001		3.93 (2.2, 7.04)	<0.001
Unfavourable	1.67 (0.80, 3.51)	0.17		2.88 (1.51, 5.49)	0.001		5.63 (3.1, 10.2)	<0.001
Unweighted lifestyle								
Favourable	Reference			1.59 (0.91, 2.77)	0.10		2.73 (1.63, 4.54)	<0.001
Intermediate	1.00 (0.52, 1.93)	1.00		2.07 (1.19, 3.58)	0.01		3.12 (1.86, 5.24)	<0.001
Unfavourable	1.43 (0.75, 2.74)	0.28		1.73 (0.95, 3.17)	0.07		3.82 (2.25, 6.48)	<0.001
Diet index								
Favourable	Reference			2.03 (1.29, 3.20)	0.002		3.34 (2.18, 5.12)	<0.001
Unfavourable	1.45 (0.85, 2.49) 0.18	0.18		1.71 (1.03, 2.85)	0.04		3.31 (2.09, 5.23)	<0.001
Total moderate-vigorous physical activity								
Favourable	Reference			1.51 (0.91, 2.52)	0.11		2.97 (1.87, 4.71)	<0.001
Unfavourable	1.34 (0.78, 2.31)	0.28		2.26 (1.40, 3.66)	<0.001		3.62 (2.3, 5.71)	<0.001
Smoke intake								
Favourable	Reference			1.56 (1.1, 2.22)	0.01		2.62 (1.9, 3.62)	<0.001

© 2022 Feng X et al. *JAMA Network Open.*

Unfavourable	0.62 (0.16, 2.39)	0.48		1.74 (0.74, 4.06)	0.20		4.79 (2.72, 8.45)	<0.001
Alcohol consumption								
Favourable	Reference			1.68 (1.14, 2.46)	0.01		2.82 (1.98, 4.02)	<0.001
Unfavourable	0.71 (0.37, 1.36)	0.30		0.99 (0.56, 1.75)	0.98		2.01 (1.28, 3.18)	<0.001
Weight								
Favourable	Reference			1.54 (0.89, 2.66)	0.12		2.63 (1.59, 4.34)	<0.001
Unfavourable	1.27 (0.73, 2.2)	0.40		2.10 (1.27, 3.48)	0.003		3.73 (2.32, 6)	<0.001

PRS=polygenic risk score.

[a]adjusted for age, genetic composition, townsend deprivation index at recruitment, qualifications and average total household income before tax, age at menarche, menopausal status, number of live births, ever taken oral contraceptive pill, and ever used hormone-replacement therapy (HRT).

© 2022 Feng X et al. *JAMA Network Open.*

eTable 15 Combined analysis of PRS and lifestyle components on the risk of thyroid cancer in men[a]

Characteristics	PRS T1		PRS T2		PRS T3	
	HR (95%CI)	*P*	HR (95%CI)	*P*	HR (95%CI)	*P*
Weighted lifestyle						
Favourable	Reference		0.9 (0.3, 2.68)	0.85	1.89 (0.76, 4.69)	0.17
Intermediate	0.64 (0.17, 2.49)	0.52	2.14 (0.81, 5.61)	0.12	1.8 (0.68, 4.77)	0.24
Unfavourable	1.03 (0.39, 2.7)	0.96	2.2 (0.94, 5.16)	0.07	3.61 (1.61, 8.13)	0.00
Unweighted lifestyle						
Favourable	Reference		1.03 (0.38, 2.73)	0.96	2.21 (0.97, 5.06)	0.06
Intermediate	0.26 (0.06, 1.24)	0.09	1.99 (0.84, 4.7)	0.12	1.9 (0.81, 4.46)	0.14
Unfavourable	1.37 (0.54, 3.49)	0.50	1.99 (0.84, 4.71)	0.12	3.39 (1.52, 7.57)	<0.001
Diet index						
Favourable	Reference		2.04 (0.83, 5.07)	0.12	4.22 (1.85, 9.6)	<0.001
Unfavourable	1.66 (0.66, 4.16)	0.28	3.02 (1.3, 7.02)	0.01	3.45 (1.5, 7.91)	<0.001
Total moderate-vigorous physical activity						
Favourable	Reference		1.65 (0.75, 3.63)	0.22	2.36 (1.13, 4.91)	0.02
Unfavourable	1.29 (0.54, 3.1)	0.57	2.75 (1.3, 5.83)	0.01	4.27 (2.11, 8.66)	<0.001
Smoke intake						
Favourable	Reference		1.95 (1.08, 3.49)	0.03	2.62 (1.5, 4.58)	<0.001
Unfavourable	1.83 (0.53, 6.25)	0.34	2.99 (1.1, 8.13)	0.03	7.27 (3.45, 15.31)	<0.001
Alcohol consumption						
Favourable	Reference		2.72 (1.4, 5.27)	<0.001	3.35 (1.76, 6.37)	<0.001
Unfavourable	1.44 (0.59, 3.53)	0.42	0.93 (0.33, 2.65)	0.89	2.95 (1.4, 6.2)	<0.001
Weight						
Favourable	Reference		1.14 (0.46, 2.79)	0.78	2.53 (1.17, 5.44)	0.02
Unfavourable	0.83 (0.35,	0.	2.1 (0.99,	0.05	2.57 (1.23,	0.01

© 2022 Feng X et al. *JAMA Network Open.*

	2.02)	69		4.46)			5.36)	

PRS = polygenic risk score.

[a]adjusted for age, genetic composition, townsend deprivation index at recruitment, qualifications and average total household income before tax.

© 2022 Feng X et al. *JAMA Network Open.*

eTable 16 Baseline characteristics of participants of thyroid cancer in the UK biobank in the sensitivity analysis				
Variable[a]	Overall (n=378,267)[b]	Non incident TC (n=377,830)	Incident TC (n=437)	*P*
Age, median (IQR), y	57.00 (50.00, 63.00)	57.00 (50.00, 63.00)	59.00 (51.00, 63.00)	
Sex, No. (%)				<0.001
Women	195343 (51.64)	195031 (51.62)	312 (71.40)	
Men	182924 (48.36)	182799 (48.38)	125 (28.60)	
Townsend deprivation index, No. (%)				0.774
1 (least deprived)	75662 (20.00)	75578 (20.00)	84 (19.22)	
2-4	226954 (60.00)	226694 (60.00)	260 (59.50)	
5 (most deprived)	75651 (20.00)	75558 (20.00)	93 (21.28)	
Educational qualifications, No. (%)				0.325
College or University degree	127178 (33.62)	127036 (33.62)	142 (32.49)	
Secondary Education	207589 (54.88)	207336 (54.88)	253 (57.89)	
Some professional qualifications	43500 (11.50)	43458 (11.50)	42 (9.61)	
Average total household income before tax, No. (%)				0.001
Less than 18,000	65654 (17.36)	65571 (17.35)	83 (18.99)	
18,000 to 30,999	82016 (21.68)	81916 (21.68)	100 (22.88)	
31,000 to 51,999	137864 (36.45)	137679 (36.44)	185 (42.33)	
52,000 to 100,000	73039 (19.31)	72982 (19.32)	57 (13.04)	
Greater than 100,000	19694 (5.21)	19682 (5.21)	12 (2.75)	
Total fruit and vegetables intake, No. (%)				0.089
Favourable	99430 (26.29)	99299 (26.28)	131 (29.98)	
Unfavourable	278837 (73.71)	278531 (73.72)	306 (70.02)	
Fish intake				0.265
Unfavourable	285188 (75.39)	284848 (75.39)	340 (77.80)	
Favourable	93079 (24.61)	92982 (24.61)	97 (22.20)	
Processed meat and red meat intake, No. (%)				0.034
Favourable	106117 (28.05)	105974 (28.05)	143 (32.72)	
Unfavourable	272150 (71.95)	271856 (71.95)	294 (67.28)	
Whole grains intake, No. (%)				0.299
Favourable	241574 (63.86)	241284 (63.86)	290 (66.36)	

© 2022 Feng X et al. *JAMA Network Open.*

Unfavourable	136693 (36.14)	136546 (36.14)	147 (33.64)		
Refine grains intake, No. (%)				0.132	
Favourable	180717 (47.77)	180492 (47.77)	225 (51.49)		
Unfavourable	197550 (52.23)	197338 (52.23)	212 (48.51)		
Diet index, No. (%)				0.138	
Favourable	201730 (53.33)	201481 (53.33)	249 (56.98)		
Unfavourable	176537 (46.67)	176349 (46.67)	188 (43.02)		
Total moderate-vigorous physical activity, No. (%)				0.005	
Favourable	193635 (51.19)	193441 (51.20)	194 (44.39)		
Unfavourable	184632 (48.81)	184389 (48.80)	243 (55.61)		
Smoke intake, No. (%)				0.757	
Favourable	340562 (90.03)	340171 (90.03)	391 (89.47)		
Unfavourable	37705 (9.97)	37659 (9.97)	46 (10.53)		
Alcohol consumption, No. (%)				<0.001	
Favourable	262079 (69.28)	261737 (69.27)	342 (78.26)		
Unfavourable	116188 (30.72)	116093 (30.73)	95 (21.74)		
Healthy weight, No. (%)				0.002	
Favourable	166414 (43.99)	166254 (44.00)	160 (36.61)		
Unfavourable	211853 (56.01)	211576 (56.00)	277 (63.39)		
Lifestyle, No. (%)				0.672	
Favourable	139334 (36.83)	139182 (36.84)	152 (34.78)		
Intermediate	124544 (32.92)	124396 (32.92)	148 (33.87)		
Unfavourable	114389 (30.24)	114252 (30.24)	137 (31.35)		
Weighted healthy lifestyle, No. (%)				<0.001	
Favourable	140033 (37.02)	139913 (37.03)	120 (27.46)		
Intermediate	127818 (33.79)	127654 (33.79)	164 (37.53)		
Unfavourable	110416 (29.19)	110263 (29.18)	153 (35.01)		
Weighted PRS1 (15 SNPs), No. (%)				<0.001	
T1	126116 (33.34)	126036 (33.36)	80 (18.31)		
T2	126062 (33.33)	125931 (33.33)	131 (29.98)		
T3	126089 (33.33)	125863 (33.31)	226 (51.72)		

[a]Variable are numbers (percentages) except for age, which was presented as median and interquartile range.

© 2022 Feng X et al. *JAMA Network Open.*

[b]There were 378,267 participants included in the sensitivity analysis, we excluded: 1) non-European descent (n = 26,852); 2)suffering from cancer (n = 47,956); 3)missing lifestyle variable (n = 49430). Finally, we included 437 TC cases and 377,830 patients without TC.

© 2022 Feng X et al. *JAMA Network Open.*

eTable 17 Associations between healthy lifestyle and incident thyroid cancer in the sensitivity analysis[a]

Characteristics	HR (95%CI)	*P*	*P* for trend[b]
Weighted healthy lifestyle			
Favourable	Reference		<0.001
Intermediate	1.39 (1.1, 1.76)	0.01	
Unfavourable	1.83 (1.44, 2.33)	<0.001	
Unweighted healthy lifestyle			
Favourable	Reference		0.08
Intermediate	1.13 (0.9, 1.42)	0.28	
Unfavourable	1.23 (0.97, 1.55)	0.08	
Total fruit and vegetables intake			
Favourable	Reference		0.93
Unfavourable	0.95 (0.77, 1.16)	0.59	
Fish intake			
Favourable	Reference		0.98
Unfavourable	0.93 (0.74, 1.17)	0.52	
Processed meat and red meat intake			
Favourable	Reference		0.65
Unfavourable	0.96 (0.78, 1.18)	0.70	
Whole grains intake			
Favourable	Reference		0.26
Unfavourable	0.97 (0.79, 1.19)	0.76	
Refine grains intake			
Favourable	Reference		0.99
Unfavourable	0.96 (0.79, 1.16)	0.67	
Sugar drinking intake			
Favourable	Reference		0.85
Unfavourable	1.03 (0.8, 1.32)	0.85	
Diet index			
Favourable	Reference		0.47
Unfavourable	1 (0.82, 1.21)	0.99	
Total moderate-vigorous physical activity			
Favourable	Reference		NA
Unfavourable	1.28 (1.06, 1.55)	0.01	
Smoke intake			
Favourable	Reference		NA
Unfavourable	1.15 (0.84, 1.57)	0.38	
Alcohol consumption			
Favourable	Reference		0.01
Unfavourable	0.67 (0.53, 0.84)	<0.001	
Healthy weight			

© 2022 Feng X et al. *JAMA Network Open.*

Favourable	Reference		NA
Unfavourable	1.37 (1.12, 1.66)	0.002	
Weighted PRS1 (15 SNPs)[£]			
T1	Reference		<0.001
T2	1.62 (1.23, 2.15)	<0.001	
T3	2.81 (2.18, 3.63)	<0.001	

[a]Adjusted for age, sex, genetic composition, townsend deprivation index at recruitment, qualifications and average total household income before tax.

[b]*P* for trend were performed by using lifestyle factors as continuous variable.

© 2022 Feng X et al. *JAMA Network Open.*

eTable 18 Combined analysis of PRS and lifestyle components on the risk of thyroid cancer in the sensitivity analysis[a]

Characteristics	PRS T1			PRS T2			PRS T3	
	HR (95%CI)	*P*		HR (95%CI)	*P*		HR (95%CI)	*P*
Weighted healthy lifestyle								
Favourable	Reference			1 (0.58, 1.73)	0.99		2.6 (1.65, 4.08)	<0.001
Intermediate	1.29 (0.77, 2.16)	0.33		2.21 (1.39, 3.52)	<0.001		2.91 (1.86, 4.55)	<0.001
Unfavourable	1.18 (0.66, 2.09)	0.58		2.62 (1.62, 4.22)	<0.001		4.63 (2.98, 7.2)	<0.001
Unweighted healthy lifestyle								
Favourable	Reference			1.4 (0.88, 2.24)	0.16		2.63 (1.73, 4.01)	<0.001
Intermediate	0.82 (0.47, 1.43)	0.47		1.95 (1.24, 3.07)	0.003		2.95 (1.93, 4.51)	<0.001
Unfavourable	1.32 (0.79, 2.2)	0.29		1.71 (1.06, 2.76)	0.03		3.18 (2.07, 4.89)	<0.001
Diet index								
Favourable	Reference			1.79 (1.22, 2.63)	0.003		3.36 (2.36, 4.78)	<0.001
Unfavourable	1.31 (0.84, 2.03)	0.23		1.91 (1.27, 2.86)	0.001		2.94 (2.02, 4.28)	<0.001
Total moderate-vigorous physical activity								
Favourable	Reference			1.47 (0.97, 2.23)	0.07		2.66 (1.83, 3.86)	<0.001
Unfavourable	1.16 (0.75, 1.79)	0.52		2.02 (1.37, 2.99)	<0.001		3.4 (2.37, 4.9)	<0.001
Smoke intake								
Favourable	Reference			1.67 (1.25, 2.24)	<0.001		2.73 (2.09, 3.58)	<0.001
Unfavourable	1.09 (0.53, 2.28)	0.81		1.33 (0.69, 2.59)	0.39		3.83 (2.46, 5.95)	<0.001
Alcohol consumption								
Favourable	Reference			1.87 (1.36, 2.57)	<0.001		3 (2.23, 4.03)	<0.001
Unfavourable	0.91 (0.56, 1.48)	0.70		0.9 (0.55, 1.47)	0.67		2.1 (1.44, 3.06)	<0.001
Healthy weight								

Favourable	Reference			1.26 (0.8, 1.99)	0.32		2.55 (1.71, 3.82)	<0.001
Unfavourable	1.12 (0.72, 1.75)	0.62		2.11 (1.41, 3.15)	<0.001		3.35 (2.29, 4.9)	<0.001

[a]adjusted for age, sex, genetic composition, townsend deprivation index at recruitment, qualifications and average total household income before tax.

© 2022 Feng X et al. *JAMA Network Open.*

eTable 19 Associations between lifestyle component and incident thyroid cancer using competing risk analysis[a]

Characteristics	HR (95%CI)	*P*
Diet index		
Favourable	Reference	
Unfavourable	0.98 (0.78, 1.23)	0.87
Total moderate-vigorous physical activity		
Favourable	Reference	
Unfavourable	1.42 (1.15, 1.77)	0.001
Smoke intake		
Favourable	Reference	
Unfavourable	1.30 (0.89, 1.90)	0.17
Alcohol consumption		
Favourable	Reference	
Unfavourable	0.72 (0.56, 0.92)	0.01
Healthy weight		
Favourable	Reference	
Unfavourable	1.32 (1.05, 1.66)	0.02
Unweighted healthy lifestyle		
Favourable	Reference	
Intermediate	1.20 (0.93, 1.56)	0.16
Unfavourable	1.30 (0.99, 1.70)	0.06
Weighted healthy lifestyle		
Favourable	Reference	
Intermediate	1.63 (1.23, 2.16)	<0.001
Unfavourable	1.91 (1.42, 2.56)	<0.001
Weighted PRS1 (15 SNPs)		
T1	Reference	
T2	1.77 (1.28, 2.43)	<0.001
T3	2.84 (2.11, 3.82)	<0.001

[a]adjusted for age, sex, genetic composition, townsend deprivation index at recruitment, qualifications and average total household income before tax.

© 2022 Feng X et al. *JAMA Network Open.*

eTable 20 Combined analysis of PRS and lifestyle components on the risk of thyroid cancer using competing risk analysis[a]								
Characteristics	PRS T1			PRS T2			PRS T3	
	HR (95%CI)	*P*		HR (95%CI)	*P*		HR (95%CI)	*P*
Weighted healthy lifestyle								
Favourable	Reference			1.28 (0.64, 2.56)	0.49		3.08 (1.69, 5.61)	<0.001
Intermediate	1.71 (0.90, 3.25)	0.10		3.00 (1.65, 5.45)	<0.001		4.07 (2.29, 7.26)	<0.001
Unfavourable	1.57 (0.77, 3.20)	0.21		3.37 (1.84, 6.19)	<0.001		5.28 (2.95, 9.45)	<0.001
Unweighted healthy lifestyle								
Favourable	Reference			1.32 (0.76, 2.26)	0.32		2.63 (1.63, 4.24)	<0.001
Intermediate	0.70 (0.36, 1.38)	0.30		2.32 (1.41, 3.82)	<0.001		2.88 (1.77, 4.69)	<0.001
Unfavourable	1.41 (0.79, 2.54)	0.25		1.83 (1.06, 3.14)	0.03		3.22 (1.97, 5.28)	<0.001
Diet index								
Favourable	Reference			2.11 (1.34, 3.34)	0.001		3.70 (2.42, 5.65)	<0.001
Unfavourable	1.49 (0.88, 2.51)	0.13		2.18 (1.36, 3.49)	0.001		3.08 (1.95, 4.85)	<0.001
Total moderate-vigorous physical activity								
Favourable	Reference			1.39 (0.86, 2.26)	0.18		2.71 (1.76, 4.17)	<0.001
Unfavourable	1.19 (0.71, 2.00)	0.50		2.52 (1.63, 3.91)	<0.001		3.53 (2.31, 5.40)	<0.001
Smoke intake								
Favourable	Reference			1.76 (1.26, 2.45)	<0.001		2.74 (2.01, 3.74)	<0.001
Unfavourable	1.04 (0.37, 2.89)	0.94		1.90 (0.91, 3.99)	0.09		4.26 (2.50, 7.25)	<0.001
Alcohol consumption								
Favourable	Reference			2.00 (1.38, 2.90)	<0.001		3.01 (2.12, 4.28)	<0.001
Unfavourable	0.93 (0.53, 1.64)	0.81		1.10 (0.64, 1.87)	0.73		2.27 (1.47, 3.48)	<0.001
Healthy weight								

© 2022 Feng X et al. *JAMA Network Open.*

Favourable	Reference			1.63 (0.97, 2.73)	0.06		2.78 (1.73, 4.48)	<0.001
Unfavourable	1.25 (0.74, 2.12)	0.41		2.31 (1.43, 3.72)	<0.001		3.59 (2.28, 5.67)	<0.001

[a]adjusted for age, sex, genetic composition, townsend deprivation index at recruitment, qualifications and average total household income before tax.

© 2022 Feng X et al. *JAMA Network Open.*

eFigure 1 Flow diagram for the Selection of TC cases in the UK biobank

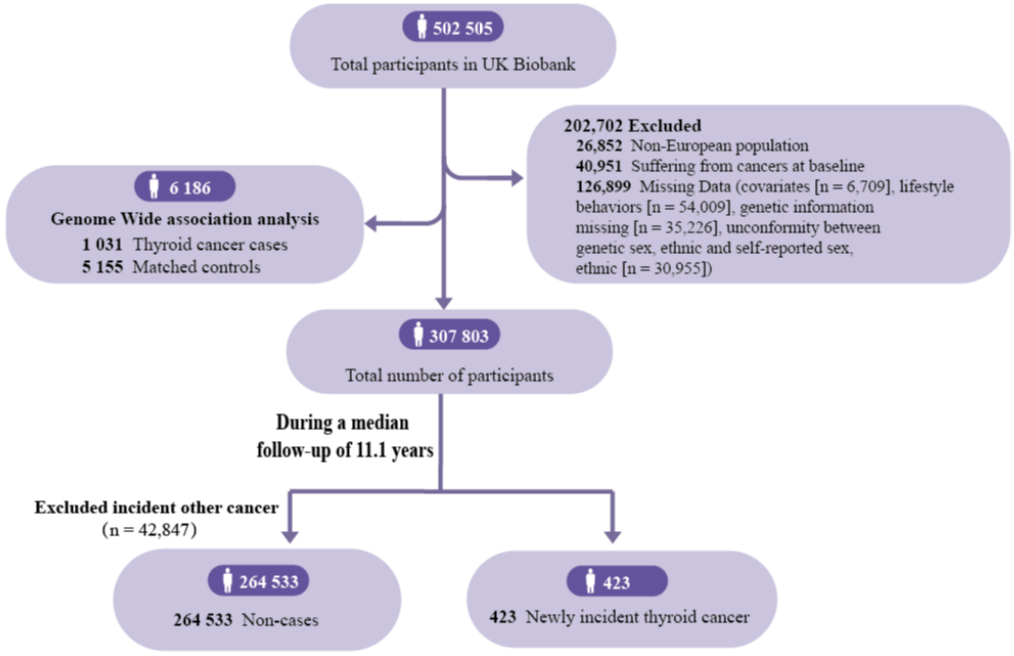

© 2022 Feng X et al. *JAMA Network Open.*

eFigure 2 Manhattan plot and QQ plot of Meta-GWAS of thyroid cancer

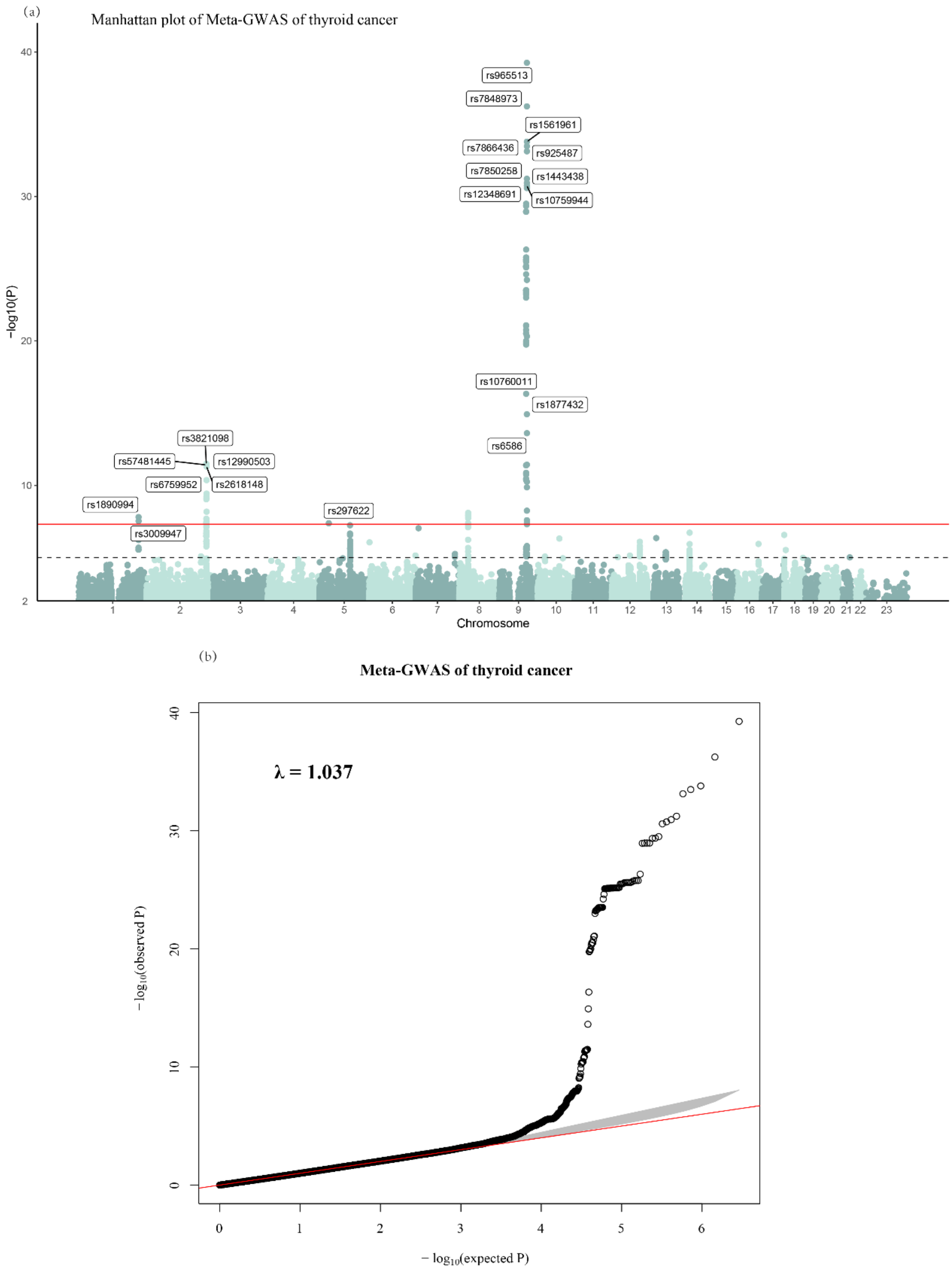

We plotted the Manhattan plot and QQ plot of Meta-GWAS of thyroid cancer. (a) Manhattan plot shows 6.5 million variants from Meta-GWAS of 1956 TC cases and 101,924 controls, the y-axis plots indicate $P<1\times10^{-2}$; (b) QQ plot with λ=1.037.

© 2022 Feng X et al. *JAMA Network Open.*

eFigure 3 ROC curve of genetic risk score

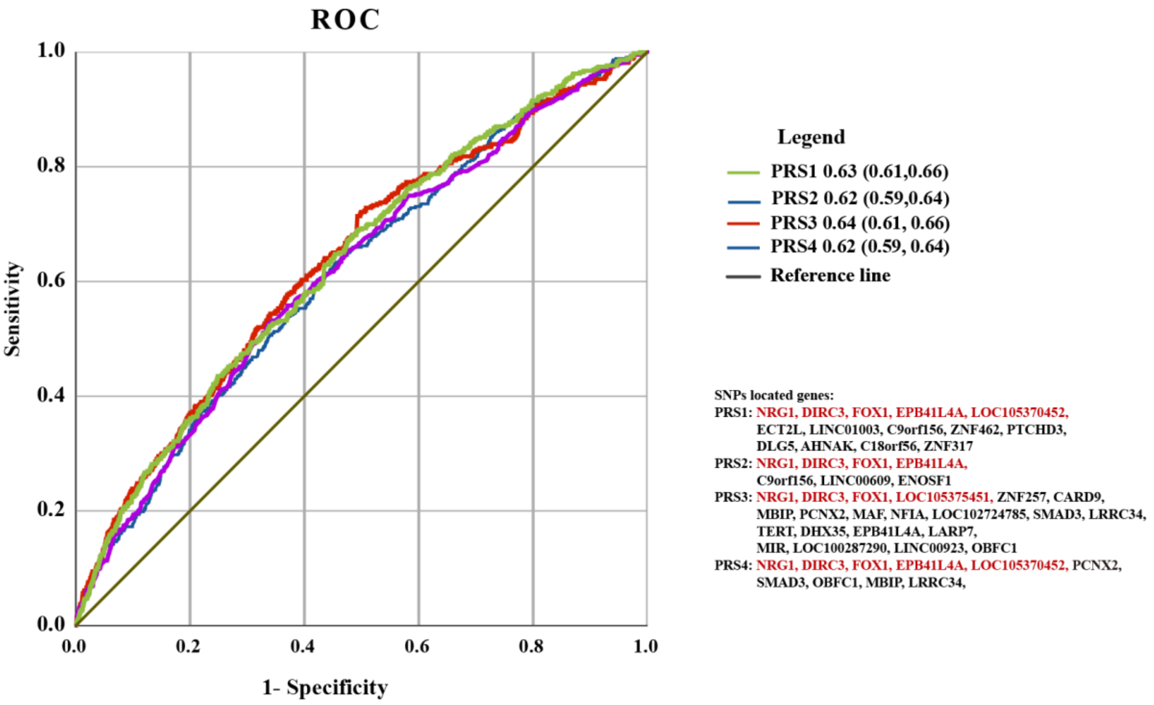

We compared the association between four PRS and TC. We obtained area under the ROC curves (AUC) of 0.63 (95%CI, 0.61 to 0.66) of PRS 1, 0.62 (95%CI, 0.59 to 0.64) of PRS 2, 0.64 (95%CI, 0.61 to 0.66) of PRS 3, and 0.62 (95%CI, 0.59 to 0.64) of PRS 4.

© 2022 Feng X et al. *JAMA Network Open.*

eFigure 4 Restricted cubic spline curve of drink score

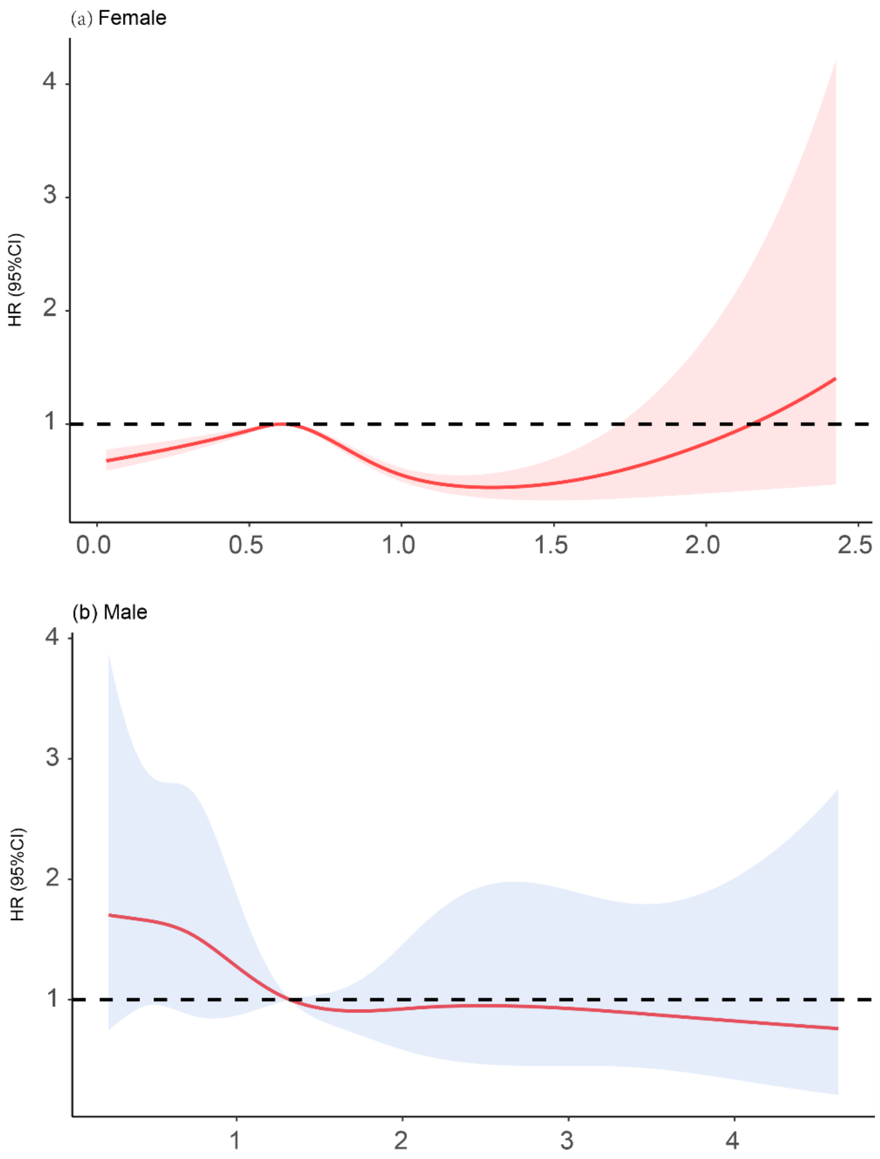

We plotted the non-linear relationship between alcohol consumption and TC using restricted cubic spline (RCS) and adjusted for age, sex, the first five genetic principal components, educational qualifications, socioeconomic status, and the average total household income before tax. (a) The non-linear relationship between drink score and TC among 122,280 females (excluded those with drink score out of 5%-95%); (b) The non-linear relationship between drink score and TC among 111,538 males (excluded those with drink score out of 5%-95%).

© 2022 Feng X et al. *JAMA Network Open.*

eReferences

1. Köhler A, Chen B, Gemignani F, Elisei R, Romei C, Figlioli G, et al. Genome-wide association study on differentiated thyroid cancer. *J Clin Endocrinol Metab*. 2013;98(10):E1674-81.

2. Gudmundsson J, Sulem P, Gudbjartsson DF, Jonasson JG, Sigurdsson A, Bergthorsson JT, et al. Common variants on 9q22.33 and 14q13.3 predispose to thyroid cancer in European populations. *Nat Genet*. 2009;41(4):460-4.

3. Gudmundsson J, Sulem P Fau - Gudbjartsson DF, Gudbjartsson Df Fau - Jonasson JG, Jonasson Jg Fau - Masson G, Masson G Fau - He H, He H Fau - Jonasdottir A, et al. Discovery of common variants associated with low TSH levels and thyroid cancer risk. *Nat Genet*. 2012;319-22(3).

4. Figlioli G, Köhler A, Chen B, Elisei R, Romei C, Cipollini M, et al. Novel genome-wide association study-based candidate loci for differentiated thyroid cancer risk. *J Clin Endocrinol Metab*. 2014;99(10):E2084-92.

5. Mancikova V, Cruz R, Inglada-Pérez L, Fernández-Rozadilla C, Landa I, Cameselle-Teijeiro J, et al. Thyroid cancer GWAS identifies 10q26.12 and 6q14.1 as novel susceptibility loci and reveals genetic heterogeneity among populations. *Int J Cancer*. 2015;137(8):1870-8.

6. Gudmundsson J, Thorleifsson G, Sigurdsson JK, Stefansdottir L, Jonasson JG, Gudjonsson SA, et al. A genome-wide association study yields five novel thyroid cancer risk loci. *Nat Commun*. 2017;8:14517.

7. Rashkin SR, Graff RE, Kachuri L, Thai KK, Alexeeff SE, Blatchins MA, et al. Pan-cancer study detects genetic risk variants and shared genetic basis in two large cohorts. Nat Commun. 2020;11(1):4423.

8. Truong T, Lesueur F, Sugier PE, Guibon J, Xhaard C, Karimi M, et al. Multiethnic genome-wide association study of differentiated thyroid cancer in the EPITHYR consortium. *Int J Cancer*.

© 2022 Feng X et al. *JAMA Network Open.*

2021;148(12):2935-46.

9. Liyanarachchi S, Gudmundsson J, Ferkingstad E, He H, Jonasson JG, Tragante V, et al. Assessing thyroid cancer risk using polygenic risk scores. *Proc Natl Acad Sci U S A*. 2020;117(11):5997-6002.

© 2022 Feng X et al. *JAMA Network Open.*
